# Molecular dynamics simulations of temperature-dependent PET binding in PETase, ThermoPETase, and FAST-PETase

**DOI:** 10.1039/d6ra00343e

**Published:** 2026-03-23

**Authors:** Athina Karaoli, Dimitris G. Mintis, Haralampos Tzoupis, Chris T. Kiranoudis, Iseult Lynch, Georgia Melagraki, Andreas Afantitis

**Affiliations:** a NovaMechanics Ltd Nicosia 1070 Cyprus afantitis@novamechanics.com; b Entelos Institute Nicosia 2102 Cyprus i.lynch@bham.ac.uk; c NovaMechanics MIKE Piraeus 18545 Greece; d School of Chemical Engineering, National Technical University Zografou 15780 Athens Greece; e School of Geography, Earth and Environmental Sciences, University of Birmingham Birmingham B15 2TT UK; f Division of Physical Sciences and Applications, Hellenic Military Academy Vari 16672 Greece; g Department of Pharmacy, Frederick University Nicosia 1036 Cyprus

## Abstract

Polyethylene terephthalate (PET) biodegradation has gained significant attention following the 2016 discovery of the PET-hydrolyzing enzyme *Is*PETase from *Ideonella sakaiensis* 201-F6. Although *Is*PETase operates under mild temperature conditions, its limited catalytic activity and poor thermal stability restrict large-scale industrial applications. To overcome these limitations, several engineered variants have been developed. In this study, the wild-type *Is*PETase was systematically compared with two engineered variants, ThermoPETase and FAST-PETase, under four temperature conditions (300, 313, 323, and 333 K) using fully detailed atomistic molecular dynamics (MD) simulations. A PET dimer was docked into the active site of each enzyme, followed by 150 ns of restrained and unrestrained MD simulations to evaluate structural stability and substrate interactions. Both engineered variants exhibited improved thermal stability relative to *Is*PETase, with FAST-PETase demonstrating the highest stability across all temperatures. Increased flexibility of the catalytic triad was observed in both engineered variants, suggesting enhanced catalytic adaptability compared to *Is*PETase. Key residues, Tyr87 and Met161, were identified as essential for stabilizing the PET dimer within the active-site cavity. At elevated temperature (333 K), *Is*PETase showed increased flexibility, leading to disrupted PET dimer binding. In contrast, ThermoPETase and FAST-PETase preserved stable PET dimer positioning at elevated temperatures, with FAST-PETase displaying the most favourable structural characteristics across all temperature conditions. Furthermore, binding energy calculations revealed a correlation between structural stability and reduced enthalpic binding energies at each enzyme's optimal temperature. Based on these analyses, the optimal temperatures for PET degradation were found to be 323 K for *Is*PETase, 333 K for ThermoPETase, and 300–313 K for FAST-PETase. These findings provide molecular-level insights into the structure–stability–activity relationships of PETase variants and highlight key determinants for the rational design of improved PET-degrading enzymes.

## Abstract 2

Comparative molecular dynamics simulations reveal temperature-dependent variations in PET dimer binding and degradation across *Is*PETase, ThermoPETase and FAST-PETase enzymes.

## Graphical abstract

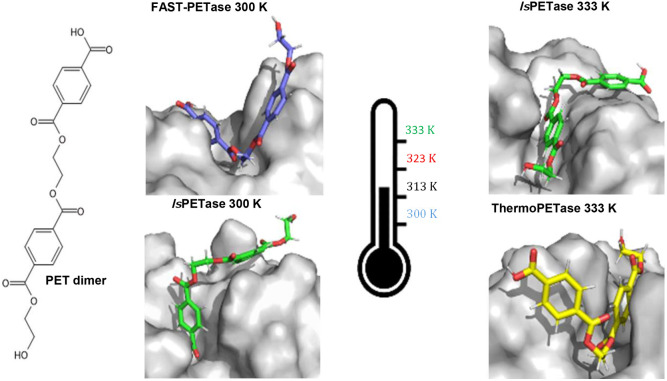

## 1 Introduction

It is estimated that by 2050, approximately 1.2 trillion tons of plastic waste will have accumulated in landfills and natural ecosystems,^1^ posing significant health risks to ecosystems and humans.^2^ The low cost and versatility of plastics^3^ make them a convenient solution for everyday human needs, with polyethylene terephthalate (PET) being the most widely produced thermoplastic polyester, due to its extensive use in the manufacture of water bottles, food packaging and textiles.^4,5^ Despite its widespread use, PET exhibits high resistance to degradation^1^ due to its physicochemical characteristics, particularly its high crystallinity and chemical stability.^5^ Environmental weathering processes, such as UV radiation, temperature fluctuations and mechanical abrasion,^6^ can lead to the fragmentation of PET into micro- and nano-plastics, contributing substantially to environmental pollution.^7^ As a result, these persistent fragments accumulate in diverse environmental compartments, including landfills^8^ and marine ecosystems,^9^ where they can persist for decades.^10^ Numerous studies^11,12^ have shown that micro- and nano-plastics can pose serious risks to both wildlife and humans through the contamination of drinking water sources and their entry into the food chain.^11,12^ The small size and large surface area of these particles also increases their potential to adsorb toxic pollutants such as perfluoroalkyl substances (PFAS) and polycyclic aromatic hydrocarbons (PAH), further intensifying their environmental impact.^13–17^

The primary strategies for managing plastic waste are currently mechanical and chemical recycling.^18^ Mechanical recycling involves the grinding of plastics to convert them into other products.^18^ However, repeated processing diminishes the material quality.^18^ Chemical recycling methods such as chemolysis and pyrolysis, break down plastics into their constituent monomers, allowing the production of new plastics or other synthetic chemicals with properties similar to virgin materials.^18–20^ However, these approaches require high energy consumption and raise environmental concerns associated with CO_2_ emissions and the generation of toxic byproducts.^19,21,22^ An innovative biotechnological approach, enzymatic biodegradation, has been developed as a more environmentally friendly alternative for plastic management, relying on microbial enzymes capable of hydrolyzing plastic polymers.^21,23^

Polyester hydrolases, cutinases and lipases are some of the key enzymes involved in the degradation of PET.^24,25^ In 2005, the first enzyme, TfH hydrolase, isolated from *Thermobifida fusca* DSM43793 was recognized for its ability to degrade PET into its monomers (*i.e.* terephthalic acid and ethylene glycol).^26^ In the following years, enzymes such as the metagenomic leaf-branch compost cutinase (LCC)^27^ and TfCut2 isolated from *Thermobifida fusca* KW3 (ref. 28) also demonstrated significant PET-degrading capabilities. Among these, an enzyme called *Is*PETase, isolated from the bacterium *Ideonella sakaiensis* 201-F6 in 2016, gained attention for its ability to degrade PET at ambient temperatures.^29^ Nevertheless, for large-scale industrial applications, enzymes must be cost-effective, highly active, and stable under specific conditions.^30^ The wild-type enzymes often fail to meet these requirements due to low activity, poor stability, and a narrow substrate range.^30^ In the case of *Is*PETase, researchers have engineered several variants through targeted mutations such as ThermoPETase,^31^ DuraPETase^32^ and FAST-PETase,^33^ each exhibiting enhanced performance compared to the wild-type. These improvements are achieved by introducing mutations that increase the rigidity of flexible loops,^34^ thereby enhancing thermostability and shifting the enzyme's optimal activity toward temperatures closer to the glass transition temperature of PET (70 °C),^31,35^ resulting in higher catalytic efficiency.

The discovery of enzymes capable of degrading PET has stimulated significant interest in elucidating the atomistic mechanisms that govern enzyme–PET interactions. Over the past five years, an increasing number of fully atomistic Molecular Dynamics (MD) simulations has been conducted to study structural flexibility, substrate binding, and conformational dynamics of PET-degrading enzymes.^36^ In addition, significant progress has been achieved through the application of hybrid Quantum Mechanics/Molecular Mechanics (QM/MM) simulations aimed at investigating catalytic pathways of PET hydrolysis at the atomistic scale.^37,38^ Table S1 in the SI provides a comprehensive overview of MD-based studies focused on PET degradation, detailing critical parameters including the specific substrate modelled, the enzyme employed, docking protocols used, molecular force fields applied, simulation duration and temperature, as well as the software platforms utilized. Annotations are also provided for studies that incorporate QM/MM methodologies to model the catalytic mechanisms. Furthermore, Table S2 in the SI presents a list of studies that integrate MD simulations with experimental data, such as X-ray crystallography^39^ or enzymatic activity assays,^40–42^ offering valuable validation and mechanistic insight, outlining similar information as in Table S1.

As presented in Tables S1 and S2, the wild-type PETase enzyme, *Is*PETase,^43–49^ is extensively studied using high-resolution crystal structures (*e.g.*, protein database (PDB) structures: 5XJH, 6EQE). These studies have provided detailed insights into the enzyme's relatively high activity at mesophilic temperatures (*i.e.*, 20 to 45 °C),^50^ and its early success in demonstrating PET hydrolysis under mild conditions.^40^ In recent years, attention has shifted toward enzymes with improved thermostability and catalytic efficiency, such as LCC,^39,51,52^ DuraPETase,^53–55^ ThermoPETase,^49,53^ FAST-PETase,^47,53,56^ and PES-H1.^51,57,58^ Notably, enzymes such as PHL7 (ref. 59 and 60) and TurboPETase,^61^ which are either derived from thermophilic organisms or designed through AI-guided engineering, have gained attention for their robust activity at elevated temperatures (*e.g.*, 65/70 °C).^59^ Similarly, Combi-PETase,^62^ a machine-learning-assisted engineered variant, has been investigated, reflecting the growing interest in predictive enzyme engineering. Additionally, enzymes such as TfCut2,^63–66^ TfCa,^67^ HiCut,^68^ and FoCut^69^ have been explored to broaden the phylogenetic and mechanistic landscape of PET biodegradation. Some studies also explore less-characterized or predicted enzymes derived from metagenomic datasets or *de novo* structure prediction (*e.g.*, AlphaFold2-based models), reflecting a trend toward integrating computational enzyme design with atomistic modeling workflows.^70–72^ In terms of substrate complexity, there has been a clear progression from early studies using minimal PET fragments (*e.g.*, MHET, BHET, 2PET)^48,73–77^ to more realistic representations such as amorphous oligomers of 4PET-9PET,^62,64,78,79^ and even crystalline polymer chains.^4^

**Fig. 1:**
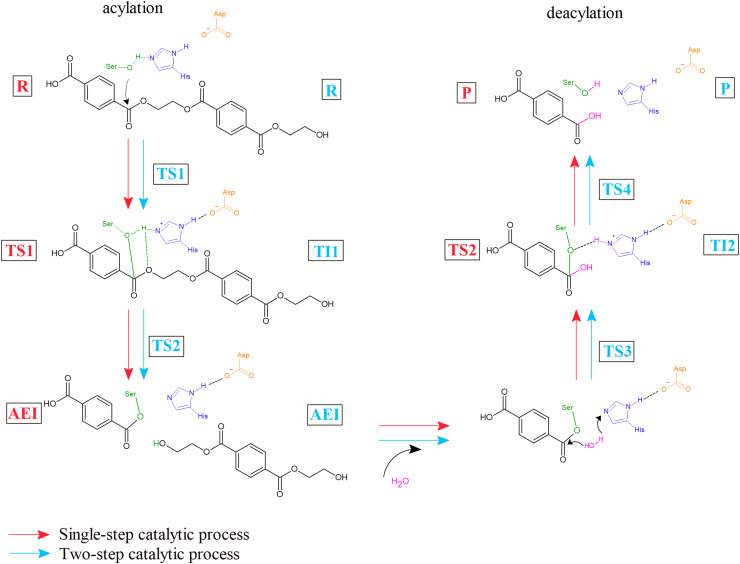
Visualization of the proposed catalytic mechanisms of a PET dimer driven by the catalytic triad, Ser (green), His (purple), and Asp (orange). The mechanism is divided into acylation and deacylation phases. The diagram also illustrates the two proposed catalytic pathways: a single-step (red) and a two-step (cyan) mechanism, as identified in QM/MM studies. Reactants (R), products (P), the acyl–enzyme intermediate (AEI), key transition states (TS), and tetrahedral intermediates (TI) are depicted to represent the full progression of the reaction.

The catalytic mechanism of *Is*PETase that promotes the enzymatic degradation of PET is driven by the catalytic triad Ser, His and Asp. Experimental investigations by Han *et al.*^80^ and Joo *et al.*^81^ proposed a two-step catalytic cycle involving acylation and deacylation, each proceeding through a tetrahedral transition state (TS), as shown in Fig. 1. In this model, the Ser residue attacks the ester bond of the PET dimer substrate, forming an acyl–enzyme intermediate (AEI) and releasing the first product, bis(2-hydroxyethyl) terephthalate (BHET), while His and Asp facilitate proton transfer within the catalytic triad. Subsequent deacylation by water regenerates the active site and releases the second product, which is terephthalic acid (TPA). This mechanism has been also supported by several QM/MM studies.^56,76,77,82^ However, other computational studies have suggested a more complex mechanism involving two distinct transition states and a short-lived tetrahedral intermediate (TI) in both catalytic steps, raising questions about whether key structures represent true intermediates or transient states.^4,44,45,57,73,74,83–85^

Tables S3 and S4 present a review of studies that computed key interatomic distances for *Is*PETase and its engineered variants, ThermoPETase and FAST-PETase, across different temperatures and PET oligomers. For *Is*PETase, most reported simulations involve oligomers of 2–4 PET monomers at 300–353 K,^43,44,53,56,76,82,83,85^ whereas studies of ThermoPETase are mainly restricted to tetramers (4-unit oligomers) of PET and those of FAST-PETase to trimer and tetramer PET.^53,56^ These structural parameters, which include distances within the catalytic triad (Ser160–His237–Asp206)††Residue numbering is the same for the three enzymes and follows the PDB files from the Protein Data Bank (PDB). and substrate-stabilizing contacts involving Tyr87 (ref. 1) and Met161,^1^ provide valuable insight into temperature-induced conformational rearrangements relevant to the catalytic hydrolysis of PET polymer. At moderate temperatures (300–313 K), triad-associated distances remain short (1.6–3.3 Å) except in da Costa *et al.*,^43^ (4.2–8.0 Å), suggesting geometries compatible with catalysis. However, at elevated temperatures (353 K), Berselli *et al.*,^53^ reported substantially larger distances, frequently exceeding 9–15 Å, indicating the disruption of the catalytic triad interactions with PET polymer and the subsequent reduction in catalytic efficiency. Temperature appears to have a limited effect on the distance between the hydroxyl oxygen atom of the side chain of Ser160 and the PET carbonyl carbon (∼2.5–3.3 Å) and stabilizing contacts with Tyr87 or Met161 (∼1.8–2.2 Å).^56,76,82,83^ Notably FAST-PETase exhibits the most robust geometries under temperature stress.^56^ Interestingly, ThermoPETase, despite its design engineering it for improved stability, demonstrates significant triad disruption at high temperature, sometimes rivaling or exceeding that observed in *Is*PETase.^53^ Collectively, these findings underscore the lack of systematic comparisons across enzyme variants at identical PET oligomer size and temperature conditions. To address this, the present study undertakes a systematic temperature-resolved comparison of *Is*PETase, ThermoPETase, and FAST-PETase in complex with dimeric PET at 300, 313, 323 and 333 K, providing a comprehensive assessment of structural dynamics and enzyme–substrate interactions across physiologically and industrially relevant temperatures. In contrast, previous atomistic studies have mainly focused on either individual PET hydrolases or on multi-enzyme systems examined at a more limited set of temperatures (Tables S1 and S2) and primarily on catalytic-triad-to-PET distances, without a unified comparison of these three widely used PETase variants.

The choice of considering a PET dimer was based on the fact that it represents the minimal oligomer that still contains a single scissile ester bond and reproduces the local chemistry of PET hydrolysis in the active site, while remaining fully compatible with the catalytic mechanism and active-site interactions reported in previous MD and QM/MM studies (Tables S1 and S2). Dimeric PET (or closely related 2MHET/2BHET fragments) has been widely adopted as a model substrate in atomistic simulations of PET hydrolases, where it has been shown to capture the key catalytic triad geometry, hydrogen-bonding network, and transition-state stabilization around the cleaved ester bond.^25,45,76,77,82^ Comparative simulations using longer oligomers (3-4PET) and amorphous or crystalline 6-9PET chains^4,52,53,85,86^ indicate that increasing chain length primarily refines distal contacts along the binding pocket and captures polymer packing effects, whereas the identity of the catalytic residues, the scissile bond environment, and the core acylation/deacylation mechanism remain consistent with PET dimer models.^44,56,82^ The temperatures selected in the current study were 300 K, 313 K, 323 K and 333 K. Temperatures around 300–313 K (27–40 °C) correspond to mesophilic and mildly elevated conditions under which *Is*PETase and other PET hydrolases have been shown to retain significant activity on PET films and oligomers, whereas temperatures between 323 and 333 K (50–60 °C) several engineered variants, such as ThermoPETase and FAST-PETase, demonstrate enhanced depolymerisation of amorphous and semi-crystalline PET while remaining structurally stable, as reported in previous experimental and computational studies.^53^ Temperatures substantially above 333–338 K (60–65 °C) would approach or exceed the glass transition temperature of PET (∼343 K) and the onset of pronounced enzyme destabilisation, where changes in polymer crystallinity, surface softening and enzyme unfolding become dominant and thus the polymer can no longer be reliably represented by a solvated PET model.^40,52,65,87–90^ Though several engineered PET hydrolases, including HotPETase, Combi-PETase, TmFae-PETase and some FAST-PETase variants, are applied experimentally at temperatures approaching or exceeding the glass transition temperature of PET, the goal of the present work is not to reproduce specific industrial process conditions, but to provide a systematic, temperature-resolved comparison of *Is*PETase, ThermoPETase and FAST-PETase under a common temperature window. Therefore, the PET dimer–enzyme complex is considered only within the 300–333 K range to provide a realistic local description of the PET–enzyme interface, capturing temperature-dependent changes in active-site geometry, hydrogen-bonding patterns and enzyme–substrate packing, while long-range effects associated with polymer crystallinity, surface morphology and multi-chain packing are beyond the scope of the present study.

Fully detailed atomistic simulations are conducted in the present study to capture enzyme–substrate interactions across a physiologically and industrially relevant temperature range (300 K, 313 K, 323 K and 333 K). Through these simulations, the effects of temperature on enzyme–substrate interactions, active site conformational flexibility, and binding stability are systematically examined. Particular attention is given to identifying temperature-induced alterations in the hydrogen-bonding network and the global conformational stability of the enzyme–substrate complexes. The structure of this work is as follows: Section 2 provides a detailed description of the computational methodology and system set up; Section 3 presents the simulation results and their analysis and Section 4 outlines the most important findings of the work and possible future research plans and directions.

## 2 Materials and methods

### 2.1 Simulation details

#### 2.1.1 Preparation of proteins and ligand

The initial coordinates of the *Is*PETase enzyme were taken from the highest-resolved X-ray crystal structure (0.92 Å) of the apoenzyme available in the Protein Data Bank (PDB), with code 6EQE.^91^ For the variants ThermoPETase and FAST-PETase, the structures were available with codes 6IJ6 (1.95 Å resolution)^31^ and 7SH6 (1.44 Å resolution),^33^ respectively. The 3D structural visualization of the secondary structure of the enzymes *Is*PETase, ThermoPETase, and FAST-PETase, along with the chemical structure of the dimer PET polymer are shown in Fig. S1. Non-terminal missing residues were added to the proteins using Chimera v1.17.3 (ref. 92) and Modeler v10.4.^93^ The water molecules and the non-standard residues were removed with pdb4amber of AmberTools23.^94^ Missing hydrogen atoms were added and adjusted to a protonation state of pH 7.0 with the histidine protonated in *δ*-position. Finally, two disulfide bonds Cys203–Cys239 and Cys273–Cys289 were formed for each protein, and the AMBER ff14SB force field^95,96^ was assigned to the protein using LEaP implemented in AmberTools23.^94^ The dimer PET polymer was constructed using Avogadro v1.98.1.^97^ The General Amber Force Field 2 (GAFF2)^98^ was assigned to the PET dimer, through LEaP using AmberTools23.^94^ The atomic partial charges were derived using the restrained electrostatic potential (RESP) method, based on electronic densities generated from single-point energy calculations at the Hartee–Fock (HF) level of theory with the 6-31G(d) basis set, using GAMESS-US software.^99^ The ligand topology was converted from AMBER to GROMACS format using the open source ACPYPE.^100–102^

#### 2.1.2 Molecular docking

Coordinate files for the receptor and ligand for the docking were prepared using AutodockTools v.4.2.^103^ The files generated (pdbqt format) included partial charges computed with the Gasteiger method for the ligand and the Kollman method for the protein. Non-polar hydrogens were not merged. Molecular docking was performed using AutoDock Vina v1.2.3.^104^ A grid was created around the receptors to define the search space for potential binding sites, with the center of the grid space determined by residues Ser160 and His237 with coordinates, in Å, *X* = −22.09, *Y* = −7.44, *Z* = 8.20 using PyMOL.^105^ The box size of the grid space for ligand docking (=24.88 Å) was calculated based on the radius of gyration of the docking compound, following the approach described by Feinstein and Brylinski.^106^ The selection of the binding orientations (poses) of the ligands (PET dimer) on the receptors (enzyme) was made by considering the highest binding affinity scores as well as the ligand's form within the pocket.

#### 2.1.3 Classical MD simulations

A summary of all systems investigated in this study is provided in Table S5. MD simulations were performed using the GROMACS v2022.3 software.^107^ The total charge of all three systems (*Is*PETase:2PET, ThermoPETase:2PET and FAST-PETase:2PET) was +6, which is neutralized with the addition of six chlorine ions. The systems were solvated in a cubic periodic box of 100 Å side length, with around 31 550 water molecules modelled with the SPC/E water model. The simulation box was approximately twice the polymer chain's end-to-end distance to prevent overlap between the polymer and its periodic images. Positional restraints were imposed on all nonhydrogen atoms (1000 kJ mol^−1^ nm^−^2) of the enzyme–PET complex structures. The systems were then energy minimized with the steepest descent algorithm allowing water molecules and ions to move freely until the maximum force converged to below 20 kJ mol^−1^ nm^−1^. Equilibration of the protein–ligand-constrained systems was conducted for 2 ns under NVT ensemble. In the NVT statistical ensemble, the system was gradually heated from 0 to 300 K using the V-rescale thermostat,^108^ followed by production MD runs conducted under NPT statistical ensemble at 300 K and 1 bar which controlled by the V-rescale thermostat^108^ and Parrinello–Rahman barostat, for a total of 150 ns.^109^ The positional restraints were maintained during energy minimization, NVT equilibration, and the first 50 ns of the NPT production runs. During this restrained simulation, only solvent molecules and ions were free to move, allowing proper solvent relaxation. After the 50 ns restrained NPT run, all restraints were released, and the systems were simulated for an additional 100 ns to capture the dynamical behavior of the enzyme–PET interactions. Four different temperatures were investigated: 300 K, 313 K, 323 K, and 333 K. In total, 12 fully detailed atomistic MD simulations were conducted. The LINCS algorithm^110^ and Particle Mesh Ewald (PME) method^111^ were used, with an integration step of 1 fs and a cutoff distance of 10 Å. The first 50 ns were conducted with restraints, while the remaining 100 ns were performed without restraints. The same protocol was applied to simulate all enzyme PET-systems.

#### 2.1.4 Asclepios KNIME nodes

**Fig. 2:**
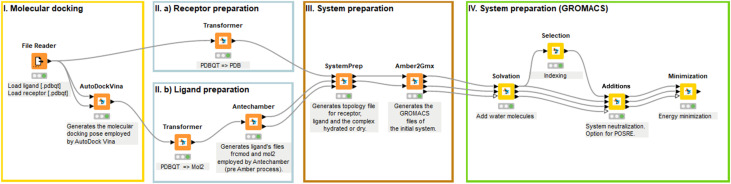
KNIME workflow with Asclepios nodes, illustrating the process from molecular docking (Step I) to protein and ligand topology preparation (Step II), followed by pre-equilibration MD steps including solvation, ion addition, and energy minimization (Steps III and IV).

KNIME is an open-source software tool that allows users to visually build custom workflows.^112^ The Asclepios nodes implemented within KNIME offer the execution of molecular docking using RxDock and AutoDock Vina while integrating AmberTools for preparing molecular topologies. In addition, Asclepios accommodates OpenMM and GROMACS for building and running all-atomistic MD simulations. The preparation of protein and ligand topologies, molecular docking and the pre-equilibration MD steps, including solvation, ions additions and energy minimization, were conducted within KNIME using Asclepios nodes. Fig. 2 presents the workflow in KNIME using Asclepios nodes. This approach not only facilitates automation but also helps minimize the risk of human error.

### 2.2 MD analysis

After completing the production MD runs of the 12 simulated systems, post-analysis was conducted on the final 10 ns equilibrated trajectory, during which no positional restraints were applied, allowing the systems to evolve freely under near-physiological conditions. Once equilibration was confirmed, further analysis was conducted, including measurements of catalytic distances and the calculation of binding energies. Plots were created using the matplotlip library in Python.^113^

#### 2.2.1 Root mean square deviation (RMSD)

The first metric used to evaluate system equilibration is the RMSD of the protein backbone. RMSD measures the extent of deviation of the protein structure over time from a reference structure (the initial docking conformation), by calculating the average displacement of the backbone atoms.^101,114–116^ The RMSD is expressed as:1
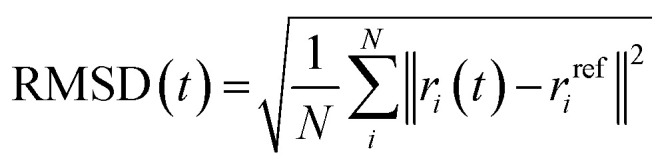
where *r*_*i*_(*t*) is the position of atom *i* at time *t* and *r*^ref^_*i*_ is the position of atom *i* at the initial frame of docking while *N* is the total number of atoms in the molecule.^116^

#### 2.2.2 Root mean square fluctuation (RMSF)

While RMSD describes the structural behavior of the whole protein, RMSF shows the structural behavior for each residue of the protein,^117,118^ by measuring the displacement of each residue relative to the reference structure (the initial docking conformation).^115^ Even in systems with similar RMSD values, the RMSF values can differ in specific regions, indicating that flexibility may vary across different areas of the protein. The RMSF is expressed as:2
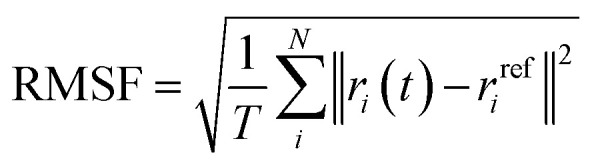
where *T* is the total time interval over which the average is calculated, *r*_*i*_(*t*) represents the position of atom *i* at time *t*, and *r*^ref^_*i*_ the reference position.^117,119^ The RMSF was calculated on C_α_ atoms (the alpha carbon, the central backbone carbon of each amino acid) relative to the initial docked conformation structure.

#### 2.2.3 Radius of gyration (*R*_g_)

*R*_g_ is a measure of protein compactness. It is defined as the root-mean-square distance of the atoms (mass-weighted) from the center of mass.^117,120^ The formula is given by:3
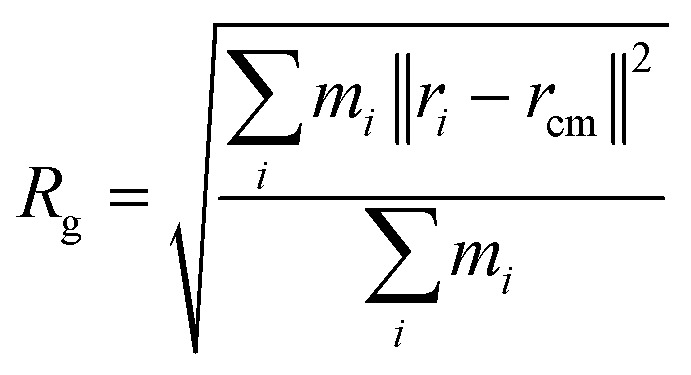
where *m*_*i*_ is the mass of atom *i* and *r*_*i*_ the position of atom *i* and *r*_cm_ is the position of the center of mass of the molecule.^116,117^

#### 2.2.4 Dictionary of secondary structure of proteins (DSSP)

To investigate the secondary structure of the protein, DSSP – an algorithm for assigning secondary structures to amino acids in protein structures based on their 3D coordinates^121^ was employed. DSSP uses geometric patterns, such as backbone torsion angles, chirality angles, and C_α_ positions, to distinguish between different structural elements.^121^ The elements analyzed include α-helix, β-bridge, β-strand, 3_10_ helix, as well as bends, turns, and loops. The π-helix was not included in this study, as it is absent in the PETase enzymes examined here. For a quantitative assessment, the percentage of each secondary structure element was calculated, and comparisons were made across different temperatures to evaluate the structural stability of the enzyme complexes studied here.

#### 2.2.5 Solvent-accessible surface area (SASA)

SASA is used to examine the structural stability and solvent exposure of the enzyme complexes studied. SASA is defined as the portion of a molecule's surface that can be contacted by solvent molecules.^122^ In this approach, the atoms on the surface of the molecule are treated as spheres of radius *R*, where *R* is the van der Waals radius of each atom, while the water molecules (with a probe radius of ∼1.4 Å) are positioned in contact with the molecule atoms in such a way that they do not overlap with any other atoms of the molecule.^101,123^ This defines the accessible surface area (ASA) of an atom, and the collection of all such contributions gives the SASA of the molecule. SASA is expressed as:^101,123,124^4
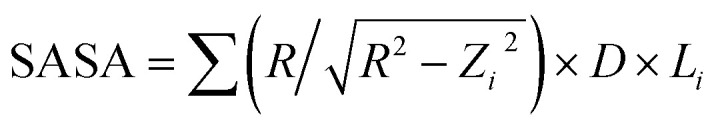
where5
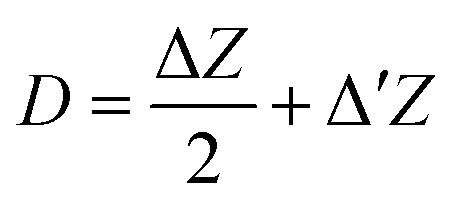
*L*_*i*_ is the length of the arc estimated on a given section *i* obtained by identifying the circles that formed the intersections of a spherical atom of van der Waals radius *R* with a set of parallel planes of predefined spacing, *Z*_*i*_ is the perpendicular distance from the center of the sphere to the section *i*, and Δ*Z* is the spacing between the sections. In addition, Δ′*Z* is defined as either Δ*Z*/2 or *R* − *Z*_*i*_, whichever is smaller. The total SASA contribution of the molecule is then obtained by summing over all such arcs.^101,123^

#### 2.2.6 Radial distribution function *g*(*r*)

This function is used to examine the average distribution of type-B atoms around a reference type-A atom. It is defined as the ratio of the average particle density of type-B atoms, *g*_B_(*r*), at a distance *r* from particle type-A and the corresponding average density in an ideal gas at the same conditions.^101^6
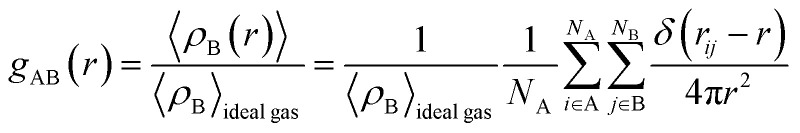


#### 2.2.7 Hydrogen bonds (HBs)

A variable for assessing the effectiveness of PET degradation is the number of HB formed between the PET dimer and the catalytic triad (Ser160–His237–Asp206), as well as with the Met161 and Tyr87 residues, which play a crucial role in stabilizing the substrate within the binding cavity. In addition, the number of HB among the residues of the catalytic triad is also an important factor, as it provides insights into the facilitation of proton transfer. For the identification of HB, the following geometric criteria were applied: (a) a donor–acceptor distance of less than 0.35 nm, and (b) a hydrogen-donor–acceptor angle of less than 30°.^101,116^

#### 2.2.8 Binding energies

The strength of PET interactions with enzymes is further quantified by performing binding free energy calculations. From the MD simulations of the enzyme:PET complexes, the binding free energies of the PET model with *Is*PETase, ThermoPETase, and FAST-PETase were estimated using the Molecular Mechanics-Poisson Boltzman Surface Area (MM-PBSA) approach. The bound protein–ligand complex is decomposed into three components: the complex, the receptor and the ligand. Snapshots of the structures are extracted at various time points during the simulation time, and these snapshots are then used for the binding free energy calculations.^125^ The MM-PBSA implicit solvent model was applied using 2000 frames, spaced every 2 ns from the trajectory, with the MMPBSA.py tool^126^ included in the AmberTools23 package integrated in GROMACS.^127^ The total binding free energy is calculated as the sum of the gas-phase interaction energy between ligand and protein (Δ*E*_MM_), the solvation energy associated to the transition from the gas-phase to the solvated state (Δ*G*_solv_) and the change in conformational entropy associated with ligand binding (eqn (6)).^125,128^ The first two terms constitute the enthalpic contribution (Δ*H*_total_), while the last represents the entropic contribution.^125,128,129^

7 Δ*G*_bind_ = Δ*E*_MM_ + Δ*G*_solv_ − *T*Δ*S*

Δ*E*_MM_ is computed from the molecular mechanics force field and consists of covalent energy (Δ*E*_covalent_), electrostatic energy (Δ*E*_elec_), and van der Waals dispersion and repulsion energy (Δ*E*_vdW_).^128,129^

8 Δ*E*_MM_ = Δ*E*_covalent_ + Δ*E*_elec_ + Δ*E*_vdW_

The covalent term includes changes in bonds (Δ*E*_bond_), angles (Δ*E*_angle_), and torsion (Δ*E*_torsion_) energies.^128,129^

9 Δ*E*_covalent_ = Δ*E*_bond_ + Δ*E*_angle_ + Δ*E*_torsion_

Δ*G*_solv_ describes the contribution of polar and non-polar interactions to the transfer of the ligand from the gas phase to solvent.^128^

10 Δ*G*_solv_ = Δ*G*_polar_ + Δ*G*_non-polar_

The polar contribution to the solvation free energy is calculated using the Poisson–Boltzmann implicit solvent model, while the non-polar contribution is calculated based on the SASA.^128^ The polar solvation component specifies the interaction energy of the solute's charge distribution in the continuum solvent and is found by evaluation of the Poisson–Boltzmann equation (PBE).^129^ The PBE is based on the fundamental Poisson equation:^125,129^

11 ∇*ε*(*r*)∇*φ*(*r*) = −4π*ρ*(*r*)

where *ε*(*r*) is the dielectric distribution function, *φ*(*r*) is the potential distribution function and *ρ*(*r*) is the fixed atomic charge density. The equation is modified to account for electrostatic interactions from ionic salt molecules in the solution, but for computational purposes, it is linearized under conditions of weak ionic strength and electric field as follows.^129^

12 ∇*ε*(*r*)∇*φ*(*r*) − *ε*_solv_*k*^2^*φ*(*r*) = −4π*ρ*(*r*)

where *k*^2^ is the Hückel parameter and it is defined as:13
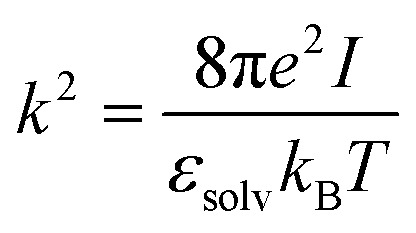
where *ε*_solv_ is the solvent dielectric constant, *k*_B_ is the Boltzmann constant and *I* is the solution ionic strength (*I* = *z*^2^*c*, where *z* is the ion charge and *c* is the ion concentration). The calculation of Δ*G*_polar_ is then defined as:^129^14
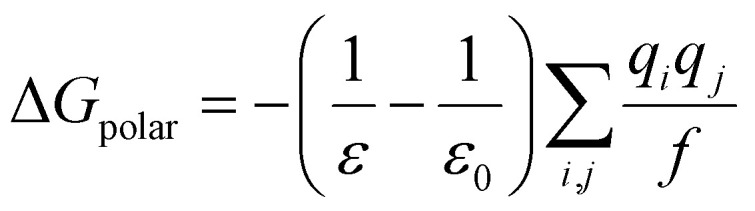
where *ε* is the internal dielectric, *ε*_0_ is the solvent dielectric constant, *q* is the partial charge and *f* is defined as:15
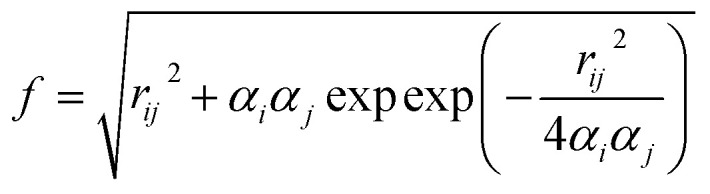
where *r*_*ij*_ is the distance between particles *i* and *j* and *α* is the effective Born radius (a constant approximately equal to the most probable distance between the nucleus and the electron in a hydrogen atom in its ground state). The non-polar solvation term measures the energy from the solute forming a cavity in the solvent and the van der Waals interactions at the cavity interface between solute and solvent.^129^ The formula is given by:

16 Δ*G*_non-polar_ = *γ* × SASA + *b*

where *γ* is the surface tension constant which describes the free energy of forming a cavity in water and *b* is the offset determined empirically. Both variables are determined empirically and set as constants for all solute molecules. These variables are defined as *γ* = 0.00542 kcal mol^−1^ Å^−2^ and *b* = 0.92 kcal mol^−1^, respectively, in the AMBER package.^129^ Lastly, the entropy term (−*T*Δ*S*) is estimated with the normal mode or quasi harmonic analysis.^125,129^

## 3 Results and discussion

### 3.1 Molecular docking

**Fig. 3:**
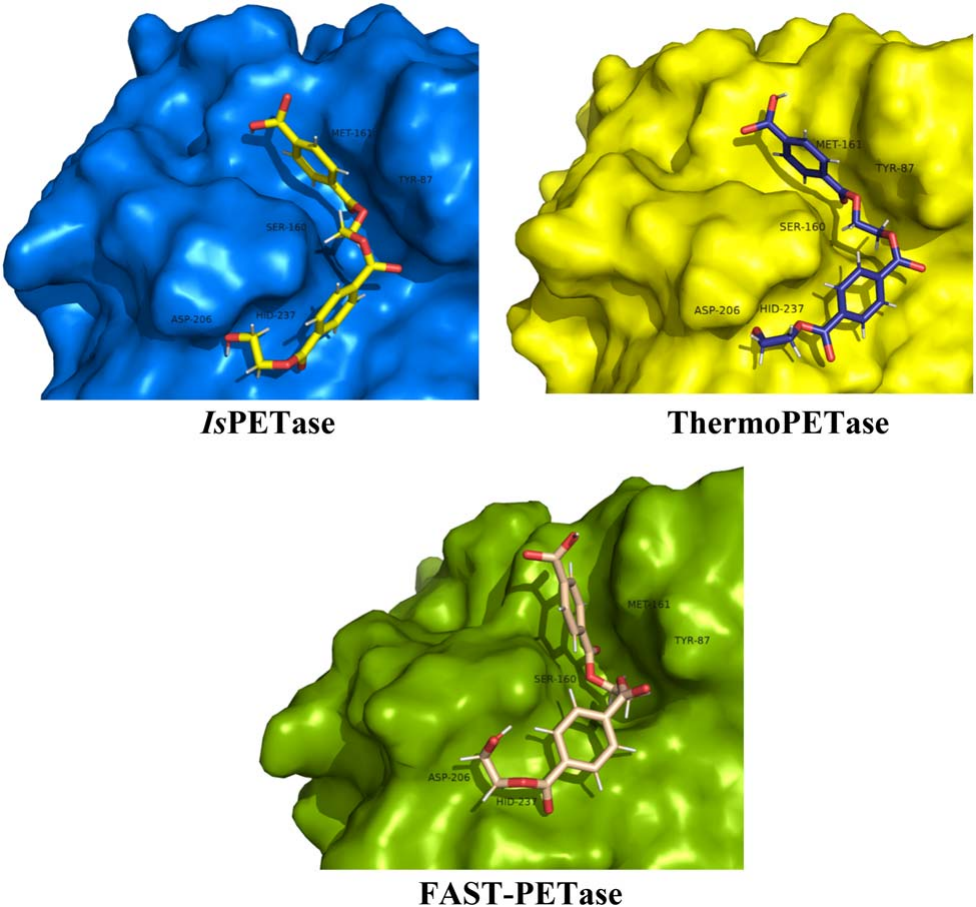
Binding modes of PET dimer complexed with the enzymes *Is*PETase, ThermoPETase, and FAST-PETase, as determined by molecular docking using AutoDock Vina.

Molecular docking was first conducted to generate plausible binding poses of the PET dimer in the active sites of *Is*PETase, ThermoPETase and FAST-PETase. The lowest-energy poses obtained with AutoDock Vina consistently placed the PET dimer in the canonical binding pocket adjacent to the Ser160–His237–Asp206 catalytic triad, in agreement with crystallographic and computational studies of PET hydrolases.^80,81^ In all three complexes, one terephthalate unit is oriented such that its carbonyl group is directed toward the nucleophilic Ser160, while the aromatic ring lies within van der Waals contact of Tyr87 and Met161, forming a hydrophobic pocket that stabilizes PET chains in PETase enzymes.^81^ Visual inspection of the docked poses (Fig. 3 and S2) shows that the overall placement of the PET dimer is qualitatively similar across the three enzymes, with only modest differences in tilt and depth of insertion into the pocket. The catalytic Ser side chain is positioned close enough to at least one ester carbonyl of the PET to enable reaction, while the His and Asp residues of the enzymes remain suitably oriented to support proton transfer. This ensures (at the docking level) that all three variants are capable of adopting geometries compatible with the first catalytic step, in line with previous structural analyses.^31,33,81^ The predicted docking scores fall within a relatively narrow range with −4.91 kcal mol^−1^ for *Is*PETase:2PET complex, −4.57 kcal mol^−1^ for ThermoPETase:2PET complex and −4.74 kcal mol^−1^ for FAST-PETase:2PET. Docking scoring functions are known to have limited quantitative reliability, and deviations of a few tenths lie well within the intrinsic uncertainty of the method.^104^ At this stage, the docking fulfilled its primary role in the present work of generating enzyme–substrate complexes that are consistent with experimental structural data, feature catalytically competent arrangements of the triad and PET dimer, and are suitable as initial configurations for subsequent MD simulations.

### 3.2 Analysis of the MD simulations

#### 3.2.1 Global structural analysis

The global structural stability of the three enzyme–substrate complexes studied here was first evaluated by analyzing the backbone RMSD and radius of gyration (*R*_g_) of enzymes at four temperatures, *i.e.*, 300, 313, 323 and 333 K (Fig. S3 and S4). These measurements provide a coarse but essential indication of whether the complexes maintain their overall fold as the temperature increases. Across the 100 ns unrestrained simulations, both RMSD and *R*_g_ exhibit stable plateau regions with only minor fluctuations around their mean values, indicating that all complexes reach and maintain conformational equilibrium without signs of progressive structural drift.

Across all simulations, backbone RMSD and *R*_g_ reach stable plateaus and remain within narrow ranges (∼0.7–1.2 Å for RMSD, 16.7–16.9 Å for *R*_g_; Table 1 and Fig. S3, S4), indicating that none of the complexes undergo global unfolding at the temperatures examined. *Is*PETase:2PET systematically shows higher RMSD than the engineered variants, in line with its lower thermal robustness reported in previous studies.^40,76,82^ ThermoPETase:2PET and FAST-PETase:2PET maintain more compact conformations with marginally lower *R*_g_ and SASA values than the wild-type enzyme (Table 1 and Fig. S5), reflecting a more compact and tightly packed surface, consistent with earlier findings.^31,33^ This increased compactness is in line with the design of these variants as thermally stabilized scaffolds and suggests a more persistent binding interface for PET at elevated temperature, as previously reported.^47,49^

**Table 1:** Average values for RMSD of the enzymes' backbone atoms, *R*_g_ of the enzymes with the respective standard errors

System	Temp (K)	RMSD (Å)	*R*_g_ (Å)
*Is*PETase:2PET	300	1.10 ± 0.06	16.84 ± 0.03
	313	1.00 ± 0.05	16.93 ± 0.04
	323	1.15 ± 0.05	16.93 ± 0.03
	333	1.21 ± 0.07	16.91 ± 0.04
ThermoPETase:2PET	300	0.83 ± 0.06	16.75 ± 0.03
	313	0.84 ± 0.07	16.77 ± 0.04
	323	0.88 ± 0.07	16.81 ± 0.04
	333	0.88 ± 0.10	16.82 ± 0.04
FAST-PETase:2PET	300	0.72 ± 0.05	16.79 ± 0.03
	313	0.84 ± 0.07	16.76 ± 0.03
	323	0.83 ± 0.06	16.80 ± 0.04
	333	0.77 ± 0.06	16.83 ± 0.03

Although RMSD and *R*_g_ clearly distinguish the global stability of the wild-type and engineered variants, it is essential to recognize that these measurements capture only global structural drift.^117^ PET hydrolases often rely on subtle, localized rearrangements, particularly within the catalytic triad and substrate-binding loops, that may not significantly alter RMSD or *R*_g_ but can strongly influence catalytic competence.^130^ Thus, while the data in Table 1 confirm that all systems remain structurally intact and that ThermoPETase and FAST-PETase exhibit better thermal robustness, these global descriptors cannot determine whether catalytically relevant geometries are preserved. For that, residue-level flexibility and catalytic distance analyses are required, which are addressed in Sections 3.2.2 and 3.2.3, respectively.

#### 3.2.2 Residual (structural) flexibility

**Fig. 4:**
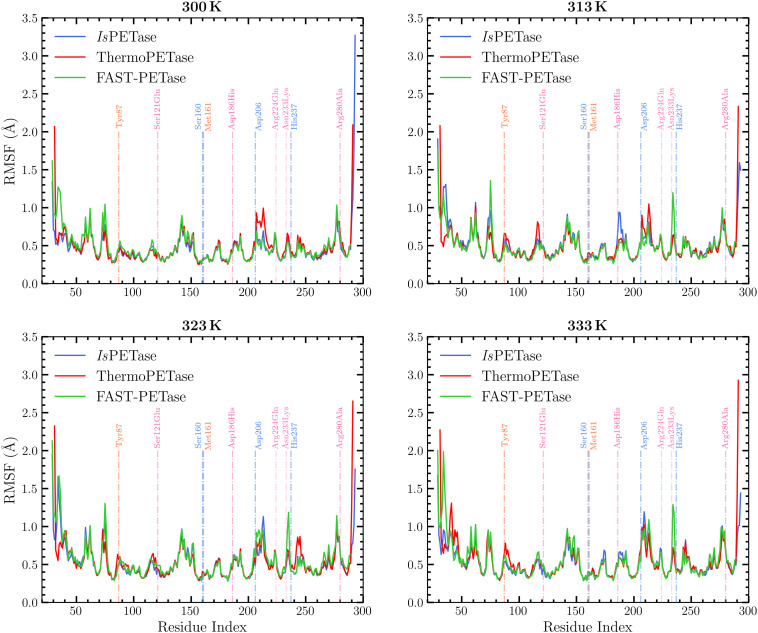
Root Mean Square Fluctuations (RMSF) of enzyme residues in the three complexes at four different temperatures. Blue dashed lines indicate the catalytic triad residues (Ser160, Asp206, and His237); pink dashed lines mark the positions of mutations in the engineered enzymes (Ser121Glu, Asp186His, Arg224Gln, Asn233Lys and Arg280Ala); and orange dashed lines indicate residues involved in PET stabilization (Tyr87 and Met161). The linear secondary structure of the enzymes is provided in Fig. S10.

The RMSF profiles of all three PETase variants are broadly similar across temperatures, with most residues fluctuating within a narrow amplitude range (Fig. 4 and Table S6), confirming the absence of large-scale unfolding. The catalytic triad residues show a modest temperature-dependent increase in flexibility, particularly for Asp206 and its surrounding loop, which becomes more mobile at higher temperatures in all three enzymes as expected due to the increase in kinetic energy, as also indicated by the free-volume analysis (Fig. S6 and S7). In the engineered variants, the catalytic loop regions are slightly more flexible than in *Is*PETase, whereas the PET-anchoring residues Tyr87 and Met161 remain relatively well stabilised across temperatures. In contrast, *Is*PETase exhibits increased flexibility of Tyr87 and Met161 at 333 K, coinciding with the loss of productive PET binding at this temperature. Mutation sites in ThermoPETase and FAST-PETase do not show a simple ‘rigid *vs.* flexible’ pattern, but overall the variants avoid the pronounced local flexibility peaks observed in the wild-type at intermediate and high temperatures. The secondary structure appears largely conserved across all the enzymes analyzed (Fig. S8). In all cases, the dominant elements are α-helices and β-strands, followed by loops, turns, and bends. Despite the presence of several mutations in the engineered enzyme variants, the overall folding of the protein does not seem to be affected. Collectively, these data indicate that engineering ThermoPETase and FAST-PETase did not simply rigidify the entire protein. Instead, it preserved global stability while allowing moderate flexibility in the catalytic loops and maintaining stable PET-anchoring residues, a combination that appears to support catalytically competent geometries under thermal stress more effectively than in *Is*PETase.

Notably, from the analyses conducted so far neither global rigidity nor residual flexibility could fully explain the catalytic behavior of the enzyme complexes studied. The overall flexibility for all complexes studied here remains broadly stable across the four temperatures examined, with no indication of global unfolding or large-scale destabilization. It is observed that the engineered variants, particularly FAST-PETase, tend to exhibit marginally higher flexibility in the catalytic triad region (especially at high temperatures) compared with the wild-type enzyme, which may allow the active site to adopt a more adaptable catalytic geometry. However, the mutation sites themselves do not display a uniform rigidity that could explain the enhanced thermostability or improved catalytic performance observed experimentally^31,33^ for these variants. In contrast, the wild-type enzyme shows increased flexibility of substrate-stabilizing residues at elevated temperatures, suggesting that excessive mobility in these regions may hinder catalytic readiness. This is confirmed by the dynamical cross-correlation matrix (DCCM) analysis shown in Fig. S9. At 333 K, low to no correlation is observed between the motions of the mutated residues and the rest of the protein, indicating increased flexibility compared with 300 K, where strong positive correlation reflects the relative stability of these regions. In the engineered enzymes, however, the correlation in the mutated regions increases at 333 K compared with 300 K, indicating enhanced rigidity of these residues, consistent with the intended stabilizing role of the mutations.

Overall, the RMSF analysis demonstrates that while engineered PETases maintain more controlled local fluctuations than the wild-type enzyme, RMSF alone cannot provide a complete mechanistic explanation for their improved catalytic properties. The results underscore the necessity of complementing the RMSF analysis with additional assessments, such as quantifying enzyme–substrate distances (presented in Section 3.2.3) and characterizing hydrogen-bonding patterns (presented in Section 3.2.4) to develop a more comprehensive understanding of the catalytic behavior in PET hydrolases under varying temperature conditions.

#### 3.2.3 Catalytic and stabilizing distances

**Fig. 5:**
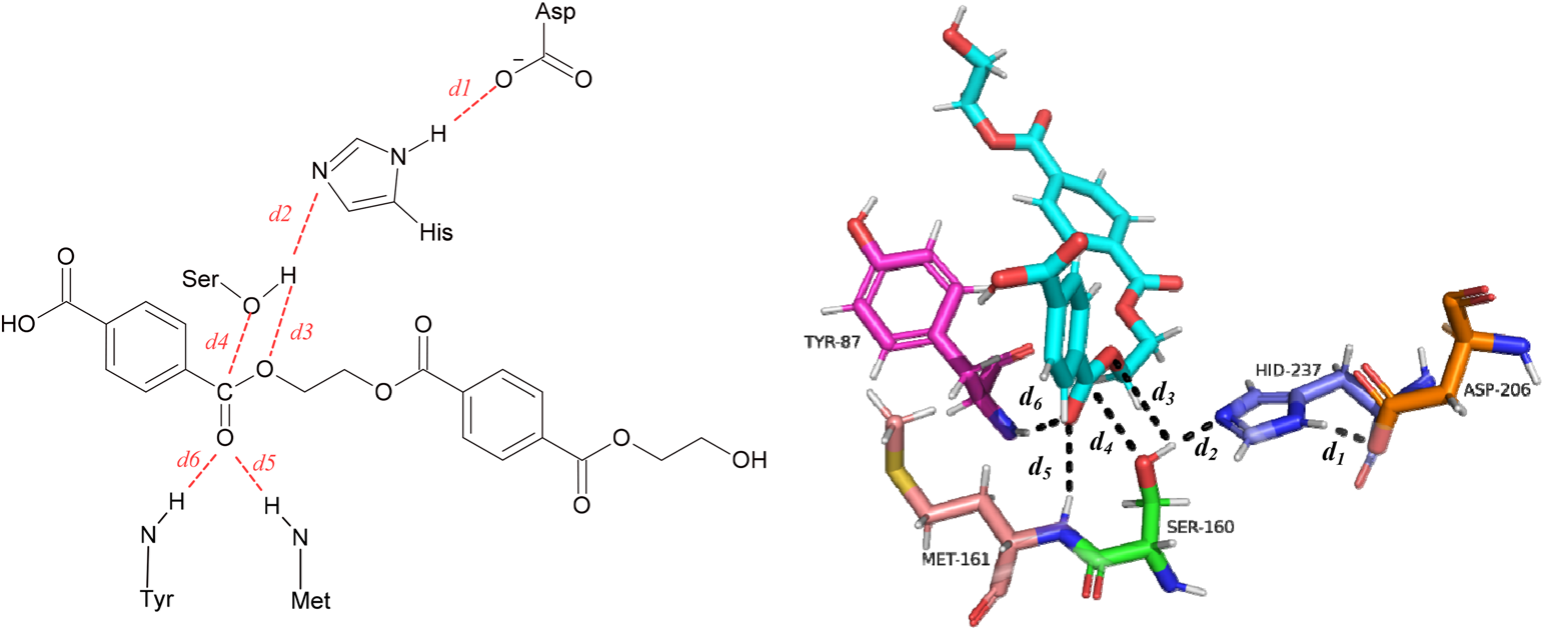
Schematic representation of the six atomic distances between key enzyme residues and PET, which are involved in catalysis and contribute to substrate stabilization, analyzed in this study. These distances comprise: the interaction between the NH of His and the negatively charged oxygen of Asp (*d*_1_); the interaction between the nitrogen of His and the hydroxyl oxygen of the catalytic Ser (*d*_2_); the distance between the catalytic serine hydroxyl oxygen and the ester oxygen of PET (*d*_3_); the distance between the catalytic serine oxygen and the PET carbonyl carbon (*d*_4_); and the distances between the NH groups of Met and Tyr (*d*_5_ and *d*_6_), respectively, and the PET carbonyl oxygen.

Key distances within the active site and between enzyme residues and the PET substrate were examined. These include (i) Ser160–His237 (*d*_1_) and His237–Asp206 (*d*_2_), which report on the geometry of the catalytic triad, (ii) Ser160–PET distances (*d*_3_, *d*_4_), which reflect the proximity of the nucleophilic hydroxyl to the scissile ester bond, and (iii) Met161–PET and Tyr87–PET distances (*d*_5_, *d*_6_), associated with substrate stabilization within the binding cleft. All distances are presented in Fig. 5 and summarized in Table S7.

**Fig. 6:**
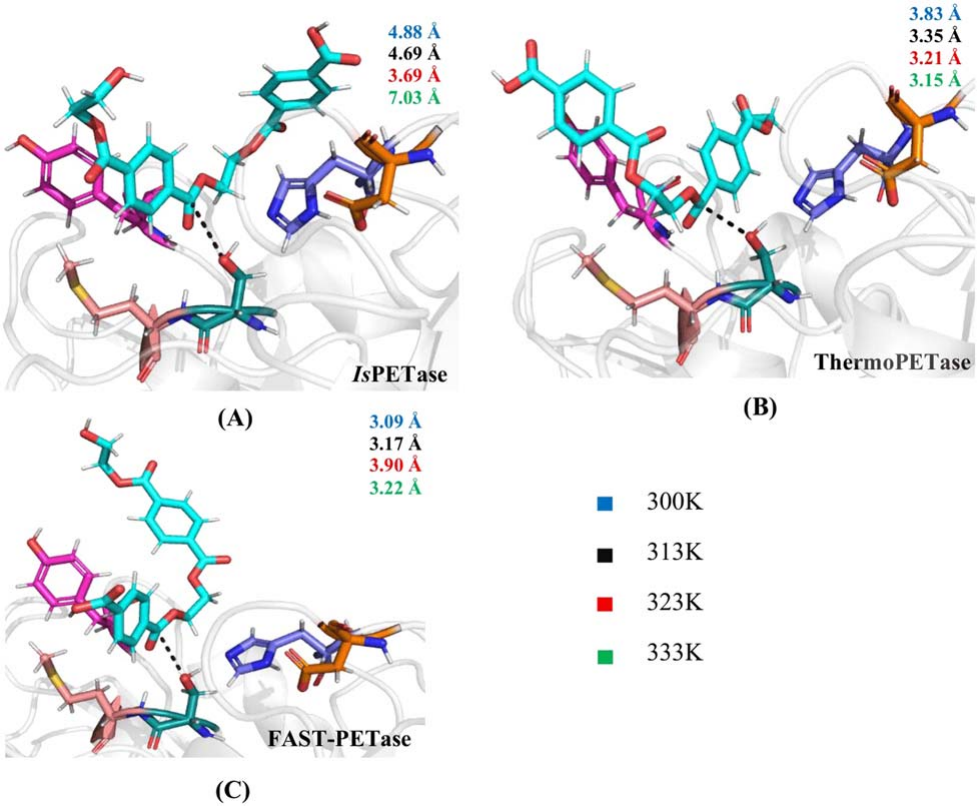
Comparison of catalytic distances in (A) *Is*PETase, (B) ThermoPETase, and (C) FAST-PETase. For each enzyme, the average distance between the catalytic serine hydroxyl oxygen and the PET carbonyl carbon is shown, with colors indicating the different simulation temperatures. These temperature-dependent changes in catalytic geometry help identify the most favorable temperature for effective PET degradation.

Fig. 6 compares the catalytic and stabilising distances in *Is*PETase, ThermoPETase and FAST-PETase across the simulated temperatures. In *Is*PETase, catalytically competent Ser–PET and Tyr87–PET distances are sampled most clearly at 323 K, where the nucleophile and oxyanion-stabilising Tyr87 adopt a more compact arrangement around the PET dimer, whereas at 300 K the substrate remains further from Ser160 and the Ser160–His237 separation is larger, indicating a less pre-organised catalytic geometry. Previous QM/MM studies^82,83^ have reported more compact catalytic distances for *Is*PETase at 300 K therefore, the present MD results should only be considered as a qualitative indication of temperature-dependent pre-organization. At 333 K, PET–Tyr87 and PET–Met161 distances increase and Ser–PET separations expand, consistent with reduced stabilisation of the substrate and temperature-induced destabilisation of substrate-orienting residues as reported previously for *Is*PETase.^43^ In ThermoPETase, catalytic and stabilising distances are systematically shorter than in the wild-type across the temperature range as confirmed from the time evolution of the distance between Ser and the ester bond of PET (Fig. S11) and become most favourable at 333 K, where Tyr87–PET and Met161–PET contacts (*d*_6_ = 2.12 Å and *d*_5_ = 2.58 Å) remain within hydrogen-bonding distances and Ser160–PET (*d*_3_ = 3.70 Å, *d*_4_ = 3.15 Å) separations fall in the range typically associated with pre-reactive configurations.^48^ FAST-PETase consistently exhibits the shortest catalytic distances at all temperatures (Table S7), with particularly favourable geometries at 300–313 K, indicating a highly pre-organised active site even under mild conditions, in agreement with its experimentally reported high activity in this regime.^33,53^ Unlike the *Is*PETase:2PET complex, PET in both ThermoPETase:2PET and FAST-PETase:2PET complexes remains stably positioned within the binding pocket at all temperatures examined. This observation is consistent with the distance distribution (Fig. S12) between the hydroxyl oxygen of the catalytic Ser and the ester carbon, which reveals a more favorable catalytic geometry for the engineered enzymes compared to *Is*PETase across all temperatures, particularly at 333 K, with modal distances of 7.12 Å for the wild-type, 2.97 Å for ThermoPETase, and 3.02 Å for FAST-PETase.

To gain further insight into how closely PET interacts with the enzymes, the radial distribution function *g*(*r*) was used to examine the spatial distribution of water molecules around the PET substrate (Fig. S13). At the mild temperature of 300 K, the function shows that water molecules are located closer to PET in the wild-type enzyme systems, with a peak at short distances from PET, indicating that water penetrates between PET and the protein pocket. In contrast, for the engineered enzymes, water molecules are distributed at larger distances from PET, reflecting a more favorable positioning and configuration of the substrate within the active site, where water molecules are largely excluded. At 333 K, the function confirms that PET tends to move away from the wild-type protein, resulting in increased water density near the PET, whereas the distribution around the engineered enzymes remains largely unchanged, consistent with the distances reported in Table S7.

Several studies have employed hybrid QM/MM approaches^44,82,83,85^ for reporting catalytic distances in PETases which directly sample reactive conformations and often yield shorter Ser160–PET/His237–Ser160 distances than those obtained from classical MD simulations. Consequently, quantitative comparisons with previous works must be made cautiously, particularly given differences in simulated temperature and substrate length (monomer/dimer/tetramer). Nevertheless, the trends observed here, such as the diminished catalytic organization at high temperature in the wild-type and improved geometries in engineered variants, are consistent with existing computational and experimental studies of PETase activity.^88,131^ Overall, the data presented here suggest that ThermoPETase maintains a more favorable geometry at higher temperatures, consistent with its engineered thermostability while FAST-PETase exhibits consistently optimal catalytic distances across all temperatures, including 300 K, reflecting its enhanced structural pre-organization. However, it is important to emphasize that the geometric proximity analysis conducted here does not guarantee catalytic activity, as electronic reorganization and transition-state stabilization cannot be captured with classical MD.^36^ Distance-based analyses must therefore be interpreted using structural descriptors rather than mechanistic predictors.

#### 3.2.4 Hydrogen bonding analysis

The computation of hydrogen bonds (HBs) between the PET dimer and key residues (Tyr87, Met161 and Ser207) was conducted to examine substrate (PET polymer) stabilization and catalytic behavior in the binding pocket (Table 2). The results show clear differences among the engineered variants and highlight limits of substrate anchoring. In *Is*PETase, PET–residue HBs are generally sparse and strongly temperature dependent. Tyr87–PET HBs reach only moderate occupancies at 313–323 K and essentially vanish at 300 and 333 K, while Met161–PET and Ser207–PET HBs remain weak or intermittent across the entire range. This pattern indicates that PET anchoring in the wild-type enzyme is transient and easily perturbed by temperature, consistent with previous MD studies that reported substrate destabilisation and drift from the binding pocket at elevated temperatures.^53,131^ In ThermoPETase, PET–residue HBs are moderately enhanced relative to *Is*PETase, particularly at higher temperatures. Met161–PET contacts become more frequent as temperature increases, and Tyr87–PET HBs are markedly more populated at 333 K than in the wild-type, indicating that the engineered mutations reinforce substrate stabilisation in the binding pocket under thermal stress. This behaviour aligns with the original design rationale for ThermoPETase as a thermally improved PETase variant.^31^ The most robust and consistent hydrogen-bonding pattern emerges in FAST-PETase. Across all temperatures, Tyr87–PET HBs exceed 50% occupancy, indicating persistent anchoring. Met161–PET HBs are also frequent, particularly at lower temperatures (300 K and 313 K), where they surpass those observed in ThermoPETase. Moreover, Ser207–PET HBs are observed with moderate frequency (∼17–20%) at most temperatures, except at 333 K, suggesting that Ser207 may contribute a secondary stabilizing role, complementing the primary aromatic-anchoring interactions. This suggests a tightly bound enzyme–substrate complex with FAST-PETase, consistent with previous reported results.^53^

**Table 2:** Percentage (%) of HBs that appear during the simulations. Percentages below 10% are omitted and shown as empty entries

System	Temp (K)	% of HB occurrence		
		Met161–PET	Tyr87–PET	Ser207–PET
*Is*PETase:2PET	300			
	313		36.73 ± 1.08	
	323		35.08 ± 1.07	12.39 ± 0.74
	333			
ThermoPETase:2PET	300		29.34 ± 1.02	12.14 ± 0.73
	313		47.63 ± 1.12	
	323	17.29 ± 0.85	21.39 ± 0.92	
	333	35.13 ± 1.07	56.97 ± 1.10	
FAST-PETase:2PET	300	53.12 ± 1.12	71.41 ± 1.01	17.04 ± 0.84
	313	45.58 ± 1.11	71.61 ± 1.01	20.29 ± 0.90
	323	35.38 ± 1.07	75.36 ± 0.96	19.29 ± 0.88
	333	26.64 ± 0.99	57.62 ± 1.10	

Overall, the findings presented here align well with recent mechanistic and structural studies of PET hydrolases. Earlier studies^48,74,132^ have shown that the backbone amines of Met161 and Tyr87 form persistent HBs with the PET carbonyl oxygen, thereby stabilizing the oxyanion and facilitating catalysis. In particular, umbrella-sampling QM/MM studies confirmed that these HBs are maintained throughout both acylation and deacylation steps, underscoring their mechanistic significance.^56,82^ Nevertheless, despite the improved HB patterns in the engineered variants, some limitations must be recognized. First, HB occupancy values derived from classical MD provide only a time-averaged, geometric proxy for binding strength and they do not capture full energetics, nor do they ensure that the bound configurations correspond to catalytically competent states. Second, our simulations employ a PET dimer, whereas realistic substrates in biological or industrial settings often involve longer oligomers or even polymer chains which will significantly alter binding dynamics, chain flexibility, and steric constraints therefore altering HB patterns and anchoring stability. In light of these limitations, the HB analysis should be considered as indicative of, but not definitive proof of, catalytic activity. While the high HB occupancy in FAST-PETase strongly supports a stable enzyme–substrate complex, only hybrid methods (*e.g.*, QM/MM free-energy sampling) can establish whether these configurations progress to a transition state and achieve productive catalysis.

#### 3.2.5 Enthalpic contributions to binding

**Fig. 7:**
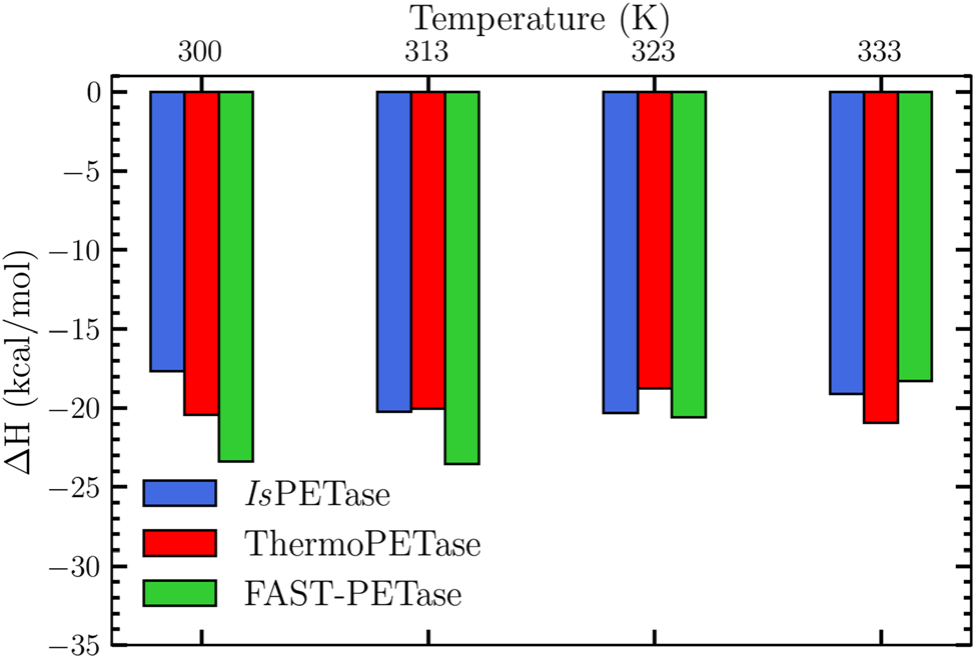
Comparison of the enthalpic energy for the 12 simulation systems (3 enzymes at 4 temperatures) as computed using the PBSA method.

To further characterize the thermodynamic driving forces of the binding process, MM-PBSA calculations were performed for all complexes at the four simulated temperatures. The resulting enthalpic binding energies are shown in Fig. 7, and the decomposition into van der Waals, electrostatic, polar solvation, and non-polar solvation terms is reported in Table S8. As is typical for protein–ligand systems, the MM-PBSA values are interpreted as qualitative trends rather than absolute binding free energies.^125,128^

For *Is*PETase, the most favourable enthalpic contribution is obtained at 323 K (Fig. 7 and Table S8), in qualitative agreement with the structural analysis, which identified this temperature as providing the shortest Ser160–PET and Tyr87–PET distances and the highest Tyr87–PET HB occupancy (Fig. 6 and Table 2). At 323 K, van der Waals interactions dominate the enthalpic term and the polar solvation penalty is reduced relative to 300 K, indicating that PET binding at this temperature requires less desolvation of the binding pocket. A similar predominance of van der Waals contributions in PETase–substrate interactions has been reported in other MM-PBSA studies of PET-binding enzymes.^133,134^ At 300 K, the less favourable enthalpic value coincides with the larger Ser160–PET and Tyr87/Met161–PET distances and low HB occupancies, indicating suboptimal conformation. At 333 K, the enthalpic term does not improve despite the higher temperature; this is consistent with the observed drift of PET away from the active site and the loss of stabilising HBs. For ThermoPETase, the most favorable enthalpic contribution is found at 333 K (−20.92 kcal mol^−1^), again in qualitative agreement with the distance and HB analyses. The short Ser160–PET, Tyr87–PET, and Met161–PET distances and the high HB occupancies observed at 333 K (Sections 3.2.3 and 3.2.4) are consistent with this enthalpic minimum, suggesting that substrate packing in the binding pocket is most effective at the highest temperature examined. These findings are in line with experimental and computational evidence indicating that ThermoPETase is designed to maintain or even improve catalytic performance at elevated temperatures relative to the wild-type enzyme.^33^ In FAST-PETase, the most favorable enthalpic values are obtained at 300 K and 313 K (−23.38 kcal mol^−1^ and −23.54 kcal mol^−1^, respectively), which are clearly more stabilizing than those of the other two enzymes at any temperature. In these conditions, van der Waals interactions again dominate the enthalpic term, reflecting tight packing of the PET dimer in the binding pocket. The shortest Ser160–PET and Tyr87/Met161–PET distances and the highest HB occupancies are also observed at these lower temperatures, indicating a strong correspondence between geometric pre-organization and enthalpic stabilization. This is consistent with computational and experimental studies which found that FAST-PETase combines enhanced thermostability with high activity under relatively mild conditions, with an active site that remains pre-organized for PET binding over a broad temperature window.^33,56^

Although the findings reported here are consistent with the structural and HB analyses presented in previous sections, several methodological limitations must be acknowledged. First, the present MM-PBSA calculations neglect explicit entropic contributions, so the reported values correspond to enthalpic components (Δ*H*_total_) rather than the total binding free energy (Δ*G*_bind_). Entropy can be a substantial factor in enzyme–substrate systems and often leads to partial enthalpy–entropy compensation.^135^ Second, endpoint methods such as MM-PBSA are known to be sensitive to molecular force-field choice, dielectric constants, and treatment of solvation, and are best employed for relative rather than absolute comparisons.^125^ Finally, the use of a PET dimer as a model substrate does not fully capture the conformational and enthalpic complexity associated with longer oligomers or semi-crystalline PET surfaces, which have been shown to exhibit different binding characteristics.^134^ Overall, the MM-PBSA analysis presented here is used only as a qualitative, supportive measure of relative substrate binding stability that complements the geometric and hydrogen-bonding analyses, rather than as a direct indicator of catalytic efficiency or optimal operating temperature. A more complete thermodynamic description would require explicit evaluation of entropic contributions and, ideally, free-energy methods or QM/MM approaches that can more rigorously capture the coupling between binding, conformational changes, and chemical reactivity.

## 4 Conclusion

In this study, fully detailed atomistic MD simulations were conducted to investigate systematically the temperature-dependent catalytic behavior of three PET-hydrolyzing enzymes: the wild-type PETase (*Is*PETase) and two engineered variants (ThermoPETase and FAST-PETase) in complex with a PET dimer substrate, across four temperatures (300, 313, 323, and 333 K). Plausible enzyme–substrate complexes were first generated by molecular docking, after which global structural stability of the three PET-hydrolyzing enzymes was evaluated through RMSD, *R*_g_ and secondary-structure analysis (DSSP). Local residue-level flexibility was evaluated through RMSF analysis, while catalytic behavior was examined through key geometric distance metrics and hydrogen-bond analysis. Finally, MM-PBSA calculations were employed to estimate enthalpic contributions to binding and to support a comparative thermodynamic interpretation across enzyme variants and temperature conditions.

The PET-hydrolyzing enzymes studied here maintained their natural fold state with temperature, while the PET dimer remained in an extended conformation within the enzyme cavity. Both engineered variants displayed significantly improved thermal stability relative to *Is*PETase, while FAST-PETase presented the greatest stability across all temperatures. Both engineered variants also displayed higher catalytic triad flexibility relative to the wild-type, corresponding to enhanced catalytic behavior. This was further supported by monitoring Tyr87 and Met161, key residues for stabilizing the PET dimer within the enzyme's active-site cavity. In *Is*PETase, elevated temperature (333 K) increased the flexibility of these residues, leading to disrupted PET positioning and destabilization, with no HB formation observed between Tyr87 or Met161 and the substrate (PET dimer). In contrast, both engineered variants maintained shorter PET–residue distances (∼2.5 Å in ThermoPETase at higher temperatures and ∼2.0 Å in FAST-PETase at lower temperatures), accompanied by a high frequency of HB formation. Importantly, the shortest PET–residue distances and highest HB occupancies correlated with the lowest enthalpic energy at each enzyme's optimal temperature. Based on these data, the potentially optimal temperatures for PET degradation are 323 K for *Is*PETase, 333 K for ThermoPETase, and 300–313 K for FAST-PETase. In all cases, PET binding is primarily found to be driven by hydrophobic interactions. Overall, these findings provide a complementary perspective to previous atomistic studies of PET hydrolases (Tables S1 and S2), which have typically focused either on individual PET hydrolases at one or two temperatures, or on multi-enzyme comparisons restricted to two temperature points and primarily analyzing catalytic-triad-to-PET distances, none of which has provided a unified, temperature-resolved comparison of the three widely used PET hydrolases examined here.

In future work, a systematic assessment of the effect of PET's degree of polymerization will be conducted for the three PET-hydrolyzing enzymes investigated here. Hybrid QM/MM calculations combined with free-energy sampling will be also employed to quantify acylation and deacylation barriers across temperatures and variants, thereby moving beyond geometric descriptors toward direct evaluation of chemical reactivity and transition-state stabilization.

## Author contributions

Conceptualization, D. G. M., A. K., I. L., A. A.; methodology, A. K., D. G. M., H. T.; formal analysis, A. K.; funding: G. M., I. L., A. A.; supervision, D. G. M., H. T., A. A.; writing-original draft preparation, A. K.; writing-review and editing, H. T., D. G. M., C. T. K., I. L., G. M. and A. A. All authors have read and agreed to the published version of the manuscript.

## Conflicts of interest

No conflict of interest to declare.

## Abbreviations

AEI: Acyl–enzyme intermediate
BHET: Bis(2-hydroxyethyl) terephthalate
DSSP: Dictionary of secondary structure of proteins
EG: Ethylene glycol
HB: Hydrogen bond
MD: Molecular dynamics
MHET: Mono(2-hydroxyethyl) terephthalate
PAH: Polycyclic aromatic hydrocarbons
PET: Polyethylene terephthalate
PFAS: Perfluoroalkyl substances
*R*_g_
: Radius of gyration
RMSD: Root mean square deviation
RMSF: Root mean square fluctuation
SASA: Solvent-accessible surface area
TI: Tetrahedral intermediate
TS: Transition state
TPA: Terephthalic acid
QM/MM: Quantum mechanics/molecular mechanics

## Supplementary Material

RA-016-D6RA00343E-s001

## Data Availability

The data supporting this article have been included as part of the supplementary information (SI). Supplementary information is available. See DOI: https://doi.org/10.1039/d6ra00343e.
