# Supplementary material for: Molecular dynamics simulations of temperature-dependent PET binding in PETase, ThermoPETase, and FAST-PETase
Source: RSC Adv. 2026 Mar 23;16(18):16050–68. doi: 10.1039/d6ra00343e (PMC13006994; doi:10.1039/d6ra00343e)
Supplement: RA-016-D6RA00343E-s001 [file RA-016-D6RA00343E-s001.pdf]

Supplementary Information (SI) for RSC Advances.
This journal is © The Royal Society of Chemistry 2026

Supplementary Material

Molecular Dynamics Simulations of Temperature-Dependent PET Binding in PETase, ThermoPETase, and FAST-PETase

Athina Karaoli,[1,4] *Dimitris G. Mintis,*[1,2] *Haralampos Tzoupis,*[1] *Chris T. Kiranoudis,*[4] *Iseult Lynch,*[2,5] *Georgia Melagraki,*[6] *Andreas Afantitis*[1,2,3,7,*]

[1]Department of ChemoInformatics, NovaMechanics Ltd., Nicosia CY-1070, Cyprus; karaoli@novamechanics.com (A.K.); mintis@novamechanics.com (D.G.M.)

[2]Entelos Institute Ltd, Larnaca 6059, Cyprus

[3]NovaMechanics MIKE, Piraeus 18545, Greece

[4]School of Chemical Engineering, National Technical University, Zografou 15780, Athens, Greece; kyr@chemeng.ntua.gr

[5]School of Geography, Earth and Environmental Sciences, University of Birmingham, Birmingham B15 2TT, United Kingdom; i.lynch@bham.ac.uk

[6]Division of Physical Sciences and Applications, Hellenic Military Academy, Vari 16672, Greece; georgiamelagraki@gmail.com

[7]Department of Pharmacy, Frederick University, Nicosia 1036, Cyprus

*Correspondence: afantitis@novamechanics.com

List of Tables

Table S1. Summary of MD simulation studies on PET-degrading enzymes. The table lists the ligands used, enzyme variants with PDB codes, and mutations tested, along with key simulation parameters, including docking procedures, force fields, simulation lengths, software packages, and any quantum calculations performed. The temperature conditions applied in each study are also indicated. 5

Table S2. Summary of studies combining (MD) simulations and experimental investigations on PET-degrading enzymes. The table provides information on the ligands used, enzyme variants with PDB codes, and mutations tested, along with key simulation parameters, including docking procedures, force fields, simulation lengths, software packages, and any quantum calculations performed. The temperature conditions employed in each study are also reported. 8

Table S3. Distances at the catalytic site, derived from the literature, for IsPETase at 300, 313 and 353K. 15

Table S4. Distances at the catalytic site, derived from the literature, for ThermoPETase and FAST-PETase at 300 and 353K. 15

Table S5. Technical details of the simulated systems investigated in this study across the three enzyme variants and different temperatures. The table includes information on simulation parameters such as force fields, simulation lengths, and the use of positional restraints. 16

Table S6. Average Root Mean Square Fluctuation (RMSF) in Å of selected enzyme residues across all systems and temperatures. The residues analyzed include the catalytic triad (Ser160, His237, Asp206), residues involved in PET stabilization (Tyr87, Met161), and the mutations introduced in the engineered variants (Ser121Glu, Asp186His, Arg224Gln, Asn233Lys and Arg280Ala). 17

Table S7. Average distances (Å) between the atoms that take part in the first phase (stage R in Figure 1) of the acylation mechanism. 18

Table S8. Molecular Mechanics-Poisson Boltzmann Surface Area (MM-PBSA) enthalpic energy (ΔH_{total}) calculations for all 12 simulated systems. Reported values include the total enthalpic energy as well as the individual energy components contributing to the enthalpic term: van der Waals interactions (ΔE_{vdW}), electrostatic interactions (ΔE_{el}), polar solvation energy calculated via the Poisson–Boltzmann model (ΔG_{polar}), and non-polar solvation energy ($\Delta G_{non\text{-}polar}$). 19

List of Figures

Figure S1. Secondary structures of the proteins: IsPETase (PDB ID: 6EQE), ThermoPETase (PDB ID: 6IJ6) and FAST-PETase (PDB ID: 7SH6) and the chemical structure of the dimer PET. Mutations are indicated in red for all three proteins. 20

Figure S2. Binding poses (orientations) for the systems IsPETase:2PET (yellow), ThermoPETase:2PET (blue) and FAST-PETase (green). 21

Figure S3. Root Mean Square Deviation (RMSD) of the enzyme backbone atoms for all 12 simulation systems, including *Is*PETase, ThermoPETase, and FAST-PETase at four temperatures (300 K, 313 K, 323 K, and 333 K). RMSD values were computed relative to the initial docked complex. 22

Figure S4. Radius of gyration (R_g) of the enzyme atoms for all 12 simulation systems, including IsPETase, ThermoPETase, and FAST-PETase at four temperatures (300 K, 313 K, 323 K, and 333 K). R_g values were calculated throughout the simulations to assess overall structural compactness. 23

Figure S5. Solvent-accessible surface area (SASA) of the enzyme atoms associated with PET binding for all 12 simulation systems, including IsPETase, ThermoPETase, and FAST-PETase at four temperatures (300 K, 313 K, 323 K, and 333 K). SASA values were monitored throughout the simulations to evaluate changes in enzyme surface exposure to the solvent, providing insights into substrate binding. 24

Figure S6. Free volume of the catalytic triad atoms as a function of time for all 12 simulation systems, including IsPETase, ThermoPETase, and FAST-PETase, simulated at four temperatures (300, 313, 323, and 333 K). Values were calculated throughout each simulation to assess temperature-dependent fluctuations and flexibility of the catalytic triad in the active site. 25

Figure S7. Time-averaged free volume of the catalytic triad atoms as a function of temperature for IsPETase, ThermoPETase, and FAST-PETase. Averages were computed over the full simulation trajectories to quantify temperature-dependent changes in active-site flexibility. 26

Figure S8. Bar plots illustrating the percentage (%) of appearance of secondary structure elements in the final frame of the simulations. Each subplot corresponds to one enzyme (*Is*PETase, ThermoPETase, and FAST-PETase), comparing results across four temperatures (300 K, 313 K, 323 K, and 333 K). 27

Figure S9. Dynamical cross-correlation matrix (DCCM) analysis of all enzyme-PET complexes at 300 K and 333 K to investigate mutation-induced coupling effects. Residue motions were

averaged over the last 10 ns of each simulation, using the docking conformation as the reference structure. he DCCM was calculated using the MD-TASK tool suite implemented in Python.[84] The color map represents correlation coefficients of residue-residue motions, where red indicates strong positive correlation (1), blue represents anti-correlation (minimum -0.4), and green/cyan corresponds to no correlation. Higher positive correlation reflects more rigid and concerted residue motions, whereas absent correlation indicates more independent and flexible movements. Dashed lines mark the positions of residues mutated in the engineered enzymes. 28

Figure S10. Secondary-structure representation of IsPETase, ThermoPETase, and FAST-PETase. Residues are shown as a linear sequence annotated with secondary-structure elements (helices, β-strands, and loops). Catalytic-triad residues (Ser160, His237, Asp206) are highlighted in green, PET-stabilizing residues (Tyr87, Met161) in orange, and engineered mutation (Ser121Glu, Asp186His, Arg224Gln, Asn233Lys and Arg280Ala) sites in pink. The secondary-structure schematics were generated using ProS^{2}Vi, a Python-based visualization tool.[85] 31

Figure S11. Time evolution of the distance between the hydroxyl oxygen of the catalytic Ser and the ester carbon of PET for all 12 simulation systems (IsPETase, ThermoPETase, and FAST-PETase) at 300, 313, 323, and 333 K. 32

Figure S12. Histogram of the distribution of the distance between the hydroxyl oxygen of the catalytic Ser residue and the ester carbon of PET for all 12 simulation systems, including IsPETase, ThermoPETase, and FAST-PETase, simulated at four temperatures (300, 313, 323, and 333 K). 33

Figure S13. Radial distribution function, g(r), describing the spatial distribution between the ester carbon of PET and the water molecules for all the systems (IsPETase, ThermoPETase, and FAST-PETase) at 300 and 333 K. The curves were smoothed using a Savitsky–Golay filter. 34

Table S1. Summary of MD simulation studies on PET-degrading enzymes. The table lists the ligands used, enzyme variants with PDB codes, and mutations tested, along with key simulation parameters, including docking procedures, force fields, simulation lengths, software packages, and any quantum calculations performed. The temperature conditions applied in each study are also indicated.

Study	Substrate	Proteins	PDB	Mutations	Docking Software	Protein force field	Ligand force field	Temp. (K)	MD length (ns)	MD software	QM/MM
Jerves et al., 2021[1]	2PET	PETase	5XH3	-	-	ff14SB	GAFF2	300	50	AMBER 18	✓
da Costa et al., 2021[2]	4PET	PETase	6EQE	-	Autodock Vina, Molegro Virtual Docker (MVD), CSD-GOLD	ff99SB	GAFF	300	500	AMBER 16	-
Boneta et al., 2021[3]	2MHET, 3MHET	PETase, LCC-ICCG	6EQE, 4EB0	-	-	ff03	GAFF	313	50	GROMACS 2018.4	✓
Feng et al., 2021[4]	2MHET	PETase	5XJH	-	Autodock Vina	CHARMM27	generated using Swissparam	298	30	CHARMM	✓
Pinto et al., 2021[5]	MHET	MHETase	6QGA	-	-	ff99SB-ILDN	GAFF	303	500	GROMACS 2018.3	✓
Aboelnga et al., 2022[6]	without ligand, 2PET	PETase, TfCut2	5XH3, 4CG1	-	Schrödinger (Glide)	ff14SB	GAFF2	300	800[a], 100[b]	AMBER 18	✓
Charupanit et al., 2022[7]	4PET	PETase	5XJH	Q119F, D112M/S238F, S238C	AutoDock4.2	GROMOS54A7	Automated Topology Builder webserver	300	100	GROMACS v5.1.2	-
James et al., 2022[8]	without ligand, 4MHET	TfCut2	4CG1	G62A/F209A	Autodock Vina	ff14SB	GAFF	300	1000	AMBER 20	-
Shrimpton-Phoenix et al., 2022[9]	HEMT	PETase	5XH3	-	AutoDock Vina	CHARMM36	generated using Swissparam	-	5	GROMACS	✓

Study	Substrate	Proteins	PDB	Mutations	Docking Software	Protein force field	Ligand force field	Temp. (K)	MD length (ns)	MD software	QM/MM
Zara et al., 2022[10]	BHET	PETase	5XJH	-	-	-	GAFF	300	150	GROMACS	-
Garcia et al., 2023[11]	3PET	PETase, FAST-PETase	6EQE, 7SH6	-	-	ff14SB	GAFF37	300	1000	AMBER 20	✓
Orlando et al., 2023[12]	without ligand, 4PET	PETase, FAST-PETase	5XJH, 7SH6	-	AutoDock4.2	ff99SB	GAFF	303, 323	100, 300	GROMACS	-
Braga et al., 2023[13]	BHET	PETase	6EQE	-	AutoDock Vina	ff14SB	GAFF	300	200	AMBER 18	-
Nelson et al., 2023[14]	BHET	PETase	5XG0	S209F/W130H, S209F/W130H/ I179Q	HADDOCK	-	CGenFF	-	3	GROMACS	-
Wang et al., 2024[15]	2MHET	BhrPETase	7EOA	-	Autodock Vina	CHARMM27	generated using Swissparam	343	30	CHARMM	✓
Berselli et al., 2024[16]	4PET	PETase, FAST-PETase, ThermoPETase, DuraPETase, HotPETase	5XJH, 7SH6, 6IJ6, 6KY5, 7QVH	-	DINC 2.0 Web Server	CHARMM36m	CGenff	303, 353	500	NAMD3	-
Burgin et al., 2024[17]	2PET	PETase	-	-	-	CHARMM36	CGenFF	310	5	CHARMM	✓
Jerves et al., 2024[18]	2PET	PETase	5XH3	D83N, D89N	-	ff14SB	GAFF2	300	600	GROMACS	✓
Pinto et al., 2024[19]	3PET	PET30	7PZJ	-	AutoDock Vina	OPLS-AA/M	OPLS-AA	300	1000	GROMACS 2021.5	-
dos Santos et al., 2024[20]	4PET	*Is*PETase	-	-	-	CHARMM	-	300	-	-	✓
Zheng et al., 2024[21]	amorph. 6PET, cryst. PET	PETase	5XJH	-	AutoDockTools 1.5.6	CHARMM36	CHARMM36	303	30	CHARMM	✓

Study	Substrate	Proteins	PDB	Mutations	Docking Software	Protein force field	Ligand force field	Temp. (K)	MD length (ns)	MD software	QM/MM
Jackering et al., 2024[22]	3PET	PES-H1, LCC	7CUV, 4EBO	-	-	ff14SB	GAFF	303	20	AMBER	✓
Xu et al., 2024[23]	4PET	PETase, ThermoPETase	5XJH, 6IJ6	-	AutoDock Vina	ff14SB	GAFF2	298, 308	1000	GROMACS 2023.2	-
James et al., 2024[24]	without ligand, 4MHET	PETase	5XH3	W130H/S209F	AutoDock Vina	ff14SB	GAFF	300	2000[a], 500[b]	AMBER 20	-
Sahihi et al., 2024[25]	9PET (bulk of 231 chains)	LCC	6THT	-	-	CHARMM36	CHARMM36	338	500	GROMACS 2021.6	-
de Oliviera et al., 2025[26]	4PET	FoCut	5XJH	-	Molegro Virtual Docker (MVD)	CHARMM36	CHARMM36	300	100	AMBER 20	-

[a]without ligand [b]complex system

Table S2. Summary of studies combining (MD) simulations and experimental investigations on PET-degrading enzymes. The table provides information on the ligands used, enzyme variants with PDB codes, and mutations tested, along with key simulation parameters, including docking procedures, force fields, simulation lengths, software packages, and any quantum calculations performed. The temperature conditions employed in each study are also reported.

Study	Substrate	Proteins	PDB	Mutations	Docking Software	Protein force field	Ligand force field	Temp. (K)	MD length (ns)*	MD software	QM/MM
Wei et al., 2014[27]	without ligand, 2PET	Tcur1278, Tcur0390	3VIS	-	GOLD	ff99SB	-	298, 353	50[a]	GROMACS 4.6	-
Then et al., 2015[28]	without ligand	TfCut2	4CG1	Ca^{2+}/Mg^{2+}	-	ff99SB	-	298	50	GROMACS 4.6	-
Fecker et al., 2017[29]	without ligand, 2PET	TfCut2, LCC, PETase	4CG1, 4EB0, 6ANE	-	Rosetta3	ff14SB	GAFF	298[a], 298[b], 323[b], 353[b]	50[a], 10[b]	AMBER 16	-
Austin et al. 2018[30]	without ligand, 4PET	PETase, TfCut2	6EQE, 4CG1	-	Glide XP (Schröding er)	CHARMM36	-	300	200[a]	NAMD 2.12	-
Knott et al., 2020 [31]	MHET	MHETase	6QZ4	-	Glide XP (Schröding er)	CHARMM36	CGenFF	303	150	NAMD 2.9	✓
Tournier et al., 2020[32]	without ligand, 3MHET	LCC	4EB0	F243I/D238C/ S283C/Y127G (LCC-ICCG)	-	ff14SB	GAFF	333	100	AMBER 16	-
Zheng et al., 2021[33]	2MHET	LCC-ICCG	6THT	-	Autodock tool	CHARMM27	generated using Swissparam	298	30	CHARMM	✓
Meng et al., 2021[34]	without ligand, 4MHET	*Is*PETase	6EQD	W159H/F229Y	Autodock Vina	CHARMM22	-	400	20[a]	NAMD 2.12	-
Liu et al.,	4PET	DuraPETase	6KY5	-	Ledock	ff14SB	GAFF	333	100	AMBER 18	-

Study	Substrate	Proteins	PDB	Mutations	Docking Software	Protein force field	Ligand force field	Temp. (K)	MD length (ns)*	MD software	QM/ MM
2022[35]											
Weigert et al., 2022[36]	4PET	PET6	7Z6B	V91T, S92A	-	CHARMM36	-	323	50	CHARMM	-
Chen et al., 2022[37]	5MHET	TfCut2	5ZOA	H184S/Q92G/ F209I/I213K	Ledock	ff14SB	GAFF	333	100	AMBER 18	✓
Yin et al., 2022[38]	$(MHET)_4$ $-CH_3$, MHET	*Is*PETase	5XG0	S92K/D157E/ R251A	Autodock Vina	-	-	298	50	GROMACS	-
Pirillo et al., 2022[39]	without ligand, 6MHET	Δ*Is*PET	6EQD	-	Ledock	ff14SB	-	303	200[a], 100[b]	GROMACS 2019.6	-
Mriqwani et al., 2022[40]	3MHET	TfCut2	4CG1	-	Glide XP (Schröding er)	-	-	-	75	Schrödinger (Desmond)	-
Haugwitzh et al., 2022[41]	MHET	TfCa,<br>MHETase	7W1J<br>6QGA	-	AutoDock Vina	-	-	310	20	AMBER 14	-
Guo et al., 2022[42]	without ligand, 4PET	PETase	6EQE	S238A, Y87E	Glide XP (Schröding er)	ff99SB-ILDN[a], OPLS-AA[b]	Parametrized via Macromodel (Schrödinger)	300	200[a], 5[b]	GROMACS 2019.3	-
Aristizabal-Lanza et al., 2022[43]	without ligand, 2MHET	HiCut	4OYY	-	Autodock Vina	AMBER14 force field	-	343	50	YASARA	-
Pfaff et al., 2022[44]	3PET	PES-H1	7CUV	-	-	ff14SB	GAFF	303	100	GROMACS 2020.4	-

Study	Substrate	Proteins	PDB	Mutations	Docking Software	Protein force field	Ligand force field	Temp. (K)	MD length (ns)*	MD software	QM/ MM
Waltmann et al., 2022[45]	without ligand	PETase	6EQE	-	-	CHARMM	-	298, 310, 320, 325, 330, 335, 340, 350	10	GROMACS 2016.3	-
Liu et al., 2022[46]	without ligand	MtCut, LCCICCG	AlphaFold, 6THT	-	GOLD	ff19SB	-	343	100	AMBER 20	-
Sevilla et al., 2023[47]	2PET	PETase	6EQE	I208V, N212A, S238Y	GOLD	ff19SB	GAFF2	300	4	AMBER 2022	-
Crnjar et al., 2023[48]	without ligand	PETase, BurPL, LCC, TfCut	7CY0, 7CW, 4EB0, 5ZOA	PETase (H214S/F218I), BurPL (H3440S/F348I), LCC (H218S/F222I), TfCut (H224S/F228I)	-	ff14SB	-	303, 308, 323, 333	500	AMBER 2020	-
Qu et al., 2023[49]	without ligand	*Is*PETase, LCC, TfCut2	6EQE, 4EB0, 4CG1	*Is*PETase (I168R/S188D, I168R/S188E)	-	ff99SB-ILDN	-	293[c], 313[c], 333[c], 353[c], 373[c], 393[c], 373[d]	20	GROMACS 5.1.4	-
Meng et al., 2023[50]	2PET	TfCut2	4CG1	L32E/S113E/T237Q	AutoDock Vina	AMBER14 force field	GAFF2	338	50	YASARA	-
Ding et al., 2023[51]	without ligand	LCCICCG	LCCICCG_ RIP structure was used	S32L/D18T/S98R/T157/ E173Q/N213P	AutoDock Vina	CHARMM	-	310	50	NAMD 2.14b2	-
Shi et al., 2023[52]	4PET	PETase	5XJH	T88I, Q119R, D220N, N246D, R260Y/S290P, Q119R/D186H, T88I/Q119R/ D220N/N246D/ R260Y/S290P (DepoPETase)	Glide XP (Schröding er)	ff19SB	GAFF2	310, 370	200 (310K), 800 (370K - HMR)	AMBER 20	-

Study	Substrate	Proteins	PDB	Mutations	Docking Software	Protein force field	Ligand force field	Temp. (K)	MD length (ns)*	MD software	QM/MM
Swidrek et al., 2023[53]	BHET, MHET, MHET(-)	CALB	1TCA		-	CHARMM36	GAFF	303	100	NAMD 2.12	✓
Pirillo et al., 2023[54]	3PET	ΔLCC	4EB0	F243T, S101N/F243T	GNINA 1.0	ff14SB	GAFF	333	200	GROMACS 2019.6	-
Zhang et al., 2023[55]	MHET	Est30	8ILT	-	-	CHARMM36	CGenFF	343	1, 5	GROMACS 2019.4	-
Richter et al., 2023[56]	EMT	PHL7	7NEI	-	Rosetta / Autodock Vina	ff19SB	GAFF	298	100	AMBER 20	-
Falkenstein et al., 2023[57]	4MHET	TfCut2	4CG1	-	-	ff99SB-ILDN	GAFF	298	100 equil. + 125 (HREX) + 300 (MetaD)	GROMACS 2018.3	-
Li et al., 2023[58]	without ligand, BHET	BsEs, ChryBHETase	AlphaFold	ΔBsEst ΔChryBHETase	AutoDock	GROMOS96 (54a7)	-	298	100	GROMACS 2016	-
Lee et al., 2023[59]	without ligand	*Is*PETase	5XJH, 6KUS, 8H5K	S121E/D186H/S242T/N246D (Is-4p), S121E/D186H/S242T/N246D/N233C/S282C/P181V/A180V/N37D/R132E/R224E/A171C/S193C (Z1-PETase)	-	ff19SB	-	323	200	GROMACS 2021.3	-
Qu et al., 2024[60]	without ligand, 2PET	PETase	6EQE	D186Q, D186H, D186N, D186A, D186V	Autodock Vina	ff99SB-ILDN	-	303[a], 303[b], 313[b], 403[b]	50	GROMACS 5.1.4	-
Ding et al., 2024[61]	without ligand, PET	PETase, V3 PETase	5XJH	K95A, R132N, K95A/R132N	Glide XP (Schrödinger)	ff14SB	GAFF	300	100, 250 (aMD)	AMBER 18	-

Study	Substrate	Proteins	PDB	Mutations	Docking Software	Protein force field	Ligand force field	Temp. (K)	MD length (ns)*	MD software	QM/ MM
Cui et al., 2024[62]	3PET	BhrPETase	7EOA	H218S/F222I/ A209R/D238K/A251C/ A281C/W104L/F243T (TurboPETase)	YASARA	ff16SB	-	338	100	AMBER 16	-
Han et al., 2024[63]	4PET	*Pp*PETase,	6QGC	Y239R/F244G/ Y250G	AutoDock Vina	-	-	300	100	GROMACS 2018.33	-
		ScPETase		A212C/T249C/ N195H/N243K							
Lu et al., 2024[64]	3PET	Est1	AlphaFold2	N213M/T215P/ S115P/Q93A/L91W	YASARA	GROMOS96 54a7	-	338	50	GROMACS 2022.3	-
Joho et al., 2024[65]	8PET	*Ps*PETase	6EQE	D186A/N233C/S282C/ A179C/S136E/S214T/ K95N (Combi-PETase)	AutoDock 4	ff14SB	GAFF2	300	1000[c], 2000[d]	AMBER 20	-
Thapa et al., 2024[66]	BHET	PET hydrolase in Streptomyces sp. W2061	SWISS-MODEL	-	AutoDock Vina	CHARMM36m	CGenFF	310	100	GROMACS	-
Zheng et al., 2024[67]	3PET	LCC-ICCG	6THT	H218Y/N248D, H218Y/N248D/ S247A	AutoDock Vina	OPLS-AA/M	generated using the LigParGen	345	20	NAMD 2.12	-
Zheng et al., 2024[68]	2BHET	LCC-ICCG	6THT	H183Y/L124G/ S29A (YGA)	Autodock Vina	ff99SB-ILDN	-	343	40	GROMACS	-
Avilan et al., 2024[69]	without ligand	*Is*PETase	6EQE/ 7QVH	HotPETase	-	CHARMM	-	300	150	AMBER 20	-
Gao et al., 2024[70]	4PET	PETase	5XJH / 8J5N	R53Q, V84L, F201I, F229Y, N233K/F, R280E, D283R, T88I/Q119R/ D220N/N246D/ R260Y/S290P (DepoPETase)	Schrödinger 2018	ff19SB	GAFF2	370	1000	AMBER 20	-

Study	Substrate	Proteins	PDB	Mutations	Docking Software	Protein force field	Ligand force field	Temp. (K)	MD length (ns)*	MD software	QM/ MM
Jackering et al., 2024[71]	9PET (bulk of 9 chains)	LCC,	4EB0	F243I/Y127G (LCCIG), F243I/Y127G/D238C/S283C (LCC-ICCG),<br><br>LCCIG (L66A/S67A/S69A), (T211V/S216A/N239L), (R143E/S145A/R151E)	-	ff14SB	GAFF2	303	1500[c], 2000[c], 100[d]	GROMACS 2020.5	-
		PES-H1	7CUV	L92F/Q94Y (PES-H1FY)<br><br>PES-H1FY (T11V/S13A/S14A), (Q26L/T27V/T28V), (T211V/S216A/N239L)							
Mamtimin et al., 2024[72]	without ligand, 2PET	TmFae-PETase	AlphaFold2 / check with 7EBO	-	AutoDock 4.2.	ff14SB	-	300[a], 328[a], 350[a]	50	AMBER 2020	-
Zhou et al., 2024[73]	MHET, MHET(-)	*Is*PETase	-	S92P/D157A (*Is*PETase^{PA})	DSDP docking	ff14SB	-	-	100	GROMACS 2024.1	-
Schreiber et al., 2024[74]	without ligand	DuraPETase	6KY5	-	-	ff99SB-ILDN	-	300	10	GROMACS 2019.3	-
Qi et al., 2024[75]	without ligand	GlacPETase	8X6V	-	-	ff99SB	-	340	20	GROMACS 2021	-
Ogura et al., 2024[76]	without ligand	PET2-7M	7EC8	H229T/F233M	-	AMBER14 force field	-	300	100	OpenMM 8.1.1	-
Numoto et al., 2024[77]	3PET	Cut190**SS	8Z2I	F77L, F81L, Ca^{2+}	-	ff99SB-ILDN	GAFF2	300	250	GROMACS	-
Miao et al., 2024[78]	MHET	BMHETase	AlphaFold2	G133S/R275D/ R247G/G406S/ A373Y/A400S	AutoDock Vina	-	-	-	500	OpenMM 7.6.0	-
Song et al.,	without ligand,	ASR1-	AlphaFold2	-	AutoDock	GROMOS96	-	298	1000	GROMACS	-

Study	Substrate	Proteins	PDB	Mutations	Docking Software	Protein force field	Ligand force field	Temp. (K)	MD length (ns)*	MD software	QM/ MM
2025[79]	BHET	PETase			Tools	(54a7)				2022.5	
Wang et al., 2025[80]	2MHET	Hydrolase 202, FAST-PETase	7QJM, 7SH6	-	Autodock Vina	CHARMM	generated using Swissparam	343	200	AMBER 18	✓
Wang et al., 2025[81]	3PET	PHL7	7NEI	E148K/T158P/S184E/H185Y (FlashPETase)	DSDP docking, Rosetta3	ff14SB	GAFF	343	70	GROMACS 2024.4	-
Guo et al., 2025[82]	2PET, 5PET, 6PET	*Is*PETase	5XG0	-	-	ff14SB	-	300	50	Q-Chem & AMBER 12	✓
Lin et al., 2025[83]	2PET	LCC-ICCG	6THT	H183Y/L202I/I208T/T153A (LCC-YITA)	DSDP docking	ff14SB-parmbsc1	-	-	50	GROMACS 2024.2	-

[a]without ligand [b]complex system [c]for wild-type [d]for mutations

Table S3. Distances at the catalytic site, derived from the literature, for *Is*PETase at 300, 313 and 353K.

Enzyme	*Is*PETase								
Temperature (K)	300						313		353
Substrate	4PET[2]	2PET[1]	3PET[11]	2PET[6]	4PET[16]	4PET[20]	2PET[3]	3PET[3]	4PET[16]
Distances (Å)									
His237 HD1 - Asp206 OD2	7.97 ± 1.37	1.62 ± 0.15	3.1 ± 0.3	1.68	3.71 ± 1.45	-	-	-	9.48 ± 2.18
Ser160 HG - His237 NE2	4.18 ± 0.78	1.76 ± 0.15	1.92 ± 0.14	1.79	3.25 ± 1.14	2.59 ± 0.09	1.84 ± 0.04	1.81 ± 0.04	15.67 ± 4.51
Ser160 HG - PET O	7.97 ± 1.37	3.78 ± 0.29	3.3 ± 0.2	-	-	3.20 ± 0.31	-	-	-
Ser160 OG - PET C	5.38 ± 0.49	3.30 ± 0.14	2.67 ± 0.09	3.09	-	2.90 ± 0.08	2.50 ± 0.03	3.05 ± 0.04	-
Met161 NH - PET O	-	3.07 ± 0.44	2.2 ± 0.2	2.1	-	-	-	-	-
Tyr87 NH - PET O	-	2.68 ± 0.57	1.94 ± 0.14	1.85	-	-	-	-	-

Enzyme	ThermoPETase		FAST-PETase		
Temperature (K)	300	353	300		353
Substrate	4PET[16]	4PET[16]	3PET[11]	4PET[16]	4PET[16]
Distances (Å)					

His237 HD1 - Asp206 OD2	1.87 ± 0.44	6.54 ± 3.06	3.0 ± 1.0	2.50 ± 1.61	8.58 ± 3.93
Ser160 HG - His237 NE2	2.10 ± 0.37	8.83 ± 4.53	1.87 ± 0.09	3.34 ± 1.04	11.53 ± 5.10
Ser160 HG - PET O	-	-	3.3 ± 0.2	-	-
Ser160 OG - PET C	-	-	2.62 ± 0.06	-	-
Met161 NH - PET O	-	-	2.2 ± 0.2	-	-
Tyr87 NH - PET O	-	-	1.87 ± 0.13	-	-

Table S4. Distances at the catalytic site, derived from the literature, for ThermoPETase and FAST-PETase at 300 and 353K.

Table S5. Technical details of the simulated systems investigated in this study across the three enzyme variants and different temperatures. The table includes information on simulation parameters such as force fields, simulation lengths, and the use of positional restraints.

System			Force Field		Conditions	Simulation Time (ns)		
No.	Protein	Ligand	Protein	Ligand	Temp. (K)	with positional restraints	without positional restraints	Total
1	*Is*PETase	2PET	ff14SB	GAFF2	300	50	100	150
2	*Is*PETase	2PET	ff14SB	GAFF2	313	50	100	150
3	*Is*PETase	2PET	ff14SB	GAFF2	323	50	100	150
4	*Is*PETase	2PET	ff14SB	GAFF2	333	50	100	150

5	ThermoPETase	2PET	ff14SB	GAFF2	300	50	100	150
6	ThermoPETase	2PET	ff14SB	GAFF2	313	50	100	150
7	ThermoPETase	2PET	ff14SB	GAFF2	323	50	100	150
8	ThermoPETase	2PET	ff14SB	GAFF2	333	50	100	150
9	FAST-PETase	2PET	ff14SB	GAFF2	300	50	100	150
10	FAST-PETase	2PET	ff14SB	GAFF2	313	50	100	150
11	FAST-PETase	2PET	ff14SB	GAFF2	323	50	100	150
12	FAST-PETase	2PET	ff14SB	GAFF2	333	50	100	150

Table S6. Average Root Mean Square Fluctuation (RMSF) in Å of selected enzyme residues across all systems and temperatures. The residues analyzed include the catalytic triad (Ser160, His237, Asp206), residues involved in PET stabilization (Tyr87, Met161), and the mutations introduced in the engineered variants (Ser121Glu, Asp186His, Arg224Gln, Asn233Lys and Arg280Ala).

System	Temp (K)	Residue

		Ser160	His237	Asp206	Tyr87	Met161	Asn121Glu	Asp186His	Arg224Gln	Asn233Lys	Arg280Ala
*Is*PETase:2PET	300	0.31	0.36	0.43	0.44	0.32	0.41	0.40	0.59	0.43	0.54
	313	0.35	0.38	0.46	0.49	0.36	0.50	0.69	0.57	0.50	0.60
	323	0.33	0.41	0.48	0.49	0.35	0.44	0.43	0.61	0.48	0.66
	333	0.36	0.42	0.55	0.53	0.35	0.45	0.48	0.69	0.50	0.69
ThermoPETase:2PET	300	0.32	0.39	0.49	0.41	0.34	0.41	0.40	0.58	0.53	0.67
	313	0.40	0.40	0.49	0.66	0.40	0.47	0.46	0.57	0.48	0.66
	323	0.41	0.44	0.51	0.57	0.39	0.44	0.45	0.62	0.54	0.64
	333	0.40	0.39	0.70	0.64	0.39	0.48	0.47	0.62	0.54	0.66
FAST-PETase:2PET	300	0.30	0.34	0.44	0.48	0.31	0.40	0.40	0.61	0.45	0.61
	313	0.32	0.37	0.47	0.43	0.33	0.46	0.44	0.61	0.71	0.60
	323	0.37	0.41	0.59	0.51	0.38	0.49	0.49	0.62	0.61	0.65
	333	0.39	0.40	0.54	0.58	0.40	0.50	0.49	0.65	0.62	0.68

Table S7. Average distances (Å) between the atoms that take part in the first phase (stage R in Figure 1) of the acylation mechanism.

Distances (Å)	*Is*PETase:2PET			
	300K	313K	323K	333K
His237 HD1 - Asp206 OD2 (***d1***)	1.81 ± 0.10	2.17 ± 0.79	1.84 ± 0.10	2.59 ± 0.37
Ser160 HG - His237 NE2 (***d2***)	3.60 ± 0.29	2.74 ± 0.83	2.69 ± 0.72	2.73 ± 0.70
Ser160 HG - PET O (***d3***)	6.20 ± 0.41	5.05 ± 1.95	4.14 ± 0.32	7.27 ± 0.90
Ser160 OG - PET C (***d4***)	4.88 ± 0.34	4.69 ± 1.77	3.69 ± 0.23	7.03 ± 1.14
Met161 NH - PET O (***d5***)	3.88 ± 0.40	5.27 ± 2.42	3.95 ± 0.46	9.07 ± 0.60
Tyr87 NH - PET O (***d6***)	4.61 ± 0.56	3.72 ± 2.11	2.45 ± 0.46	8.09 ± 0.77
	ThermoPETase:2PET			
	300K	313K	323K	333K
His237 HD1 - Asp206 OD2 (***d1***)	1.83 ± 0.10	1.85 ± 0.12	1.83 ± 0.10	1.80 ± 0.10
Ser160 HG - His237 NE2 (***d2***)	3.29 ± 0.55	2.20 ± 0.55	2.22 ± 0.69	1.96 ± 0.19
Ser160 HG - PET O (***d3***)	3.99 ± 0.23	3.69 ± 0.57	3.68 ± 0.50	3.70 ± 0.41
Ser160 OG - PET C (***d4***)	3.83 ± 0.18	3.35 ± 0.40	3.21 ± 0.30	3.15 ± 0.18
Met161 NH - PET O (***d5***)	4.11 ± 0.34	3.18 ± 0.83	2.31 ± 0.23	2.58 ± 0.33
Tyr87 NH - PET O (***d6***)	2.74 ± 0.40	2.18 ± 0.51	2.09 ± 0.22	2.12 ± 0.20
	FAST-PETase:2PET			
	300K	313K	323K	333K

His237 HD1 - Asp206 OD2 (***d1***)	1.84 ± 0.12	1.90 ± 0.15	1.85 ± 0.10	1.82 ± 0.11
Ser160 HG - His237 NE2 (***d2***)	2.09 ± 0.26	1.98 ± 0.20	2.10 ± 0.29	2.04 ± 0.38
Ser160 HG - PET O (***d3***)	3.47 ± 0.32	3.66 ± 0.30	4.37 ± 1.05	3.79 ± 0.66
Ser160 OG - PET C (***d4***)	3.09 ± 0.15	3.17 ± 0.20	3.90 ± 0.90	3.22 ± 0.35
Met161 NH - PET O (***d5***)	2.40 ± 0.26	2.50 ± 0.29	3.78 ± 1.05	2.57 ± 0.38
Tyr87 NH - PET O (***d6***)	2.00 ± 0.12	1.99 ± 0.12	2.71 ± 1.04	2.26 ± 0.47

Table S8. Molecular Mechanics-Poisson Boltzmann Surface Area (MM-PBSA) enthalpic energy (ΔH_{total}) calculations for all 12 simulated systems. Reported values include the total enthalpic energy as well as the individual energy components contributing to the enthalpic term: van der Waals interactions (ΔE_{vdW}), electrostatic interactions (ΔE_{el}), polar solvation energy calculated via the Poisson–Boltzmann model (ΔG_{polar}), and non-polar solvation energy ($\Delta G_{non\text{-}polar}$).

System	Temp (K)	Energy (kcal/mol)						
		ΔE_{vdW}	ΔE_{el}	ΔG_{polar} (PB)	$\Delta G_{non\text{-}polar}$	ΔE_{MM}	ΔG_{solv}	ΔH_{total}
	300	-31.09 ± 0.11	-14.06 ± 0.22	32.29 ± 0.21	-4.83 ± 0.01	-45.15 ± 0.24	27.47 ± 0.20	-17.68 ± 0.12
*Is*PETase:2PET	313	-28.19 ± 0.17	-13.94 ± 0.26	26.43 ± 0.26	-4.55 ± 0.02	-42.13 ± 0.33	21.88 ± 0.24	-20.25 ± 0.17
	323	-30.14 ± 0.14	-11.38 ± 0.20	25.92 ± 0.19	-4.72 ± 0.02	-41.52 ± 0.24	21.21 ± 0.18	-20.31 ± 0.16

	333	-27.64 ± 0.20	-12.10 ± 0.28	25.28 ± 0.23	-4.66 ± 0.02	-39.73 ± 0.37	20.62 ± 0.22	-19.11 ± 0.21
ThermoPETase:2PET	300	-28.03 ± 0.14	-14.51 ± 0.31	26.62 ± 0.27	-4.52 ± 0.02	-42.55 ± 0.36	22.10 ± 0.25	-20.44 ± 0.18
	313	-29.39 ± 0.16	-11.26 ± 0.26	25.13 ± 0.24	-4.53 ± 0.02	-40.65 ± 0.31	20.61 ± 0.23	-20.04 ± 0.16
	323	-31.70 ± 0.14	-13.76 ± 0.28	31.48 ± 0.24	-4.78 ± 0.01	-45.46 ± 0.36	26.70 ± 0.23	-18.76 ± 0.19
	333	-32.13 ± 0.13	-16.26 ± 0.23	32.23 ± 0.21	-4.77 ± 0.01	-48.38 ± 0.27	27.46 ± 0.21	-20.92 ± 0.13
FAST-PETase:2PET	300	-31.75 ± 0.15	-15.76 ± 0.38	28.76 ± 0.32	-4.63 ± 0.02	-47.51 ± 0.44	24.13 ± 0.30	-23.38 ± 0.19
	313	-33.48 ± 0.13	-15.75 ± 0.36	30.66 ± 0.29	-4.98 ± 0.01	-49.23 ± 0.41	25.68 ± 0.28	-23.54 ± 0.20
	323	-29.44 ± 0.19	-13.86 ± 0.31	27.19 ± 0.31	-4.48 ± 0.03	-43.30 ± 0.43	22.72 ± 0.29	-20.59 ± 0.19
	333	-29.55 ± 0.21	-12.22 ± 0.26	27.99 ± 0.23	-4.50 ± 0.02	-41.77 ± 0.40	-18.29 ± 0.24	-18.29 ± 0.24

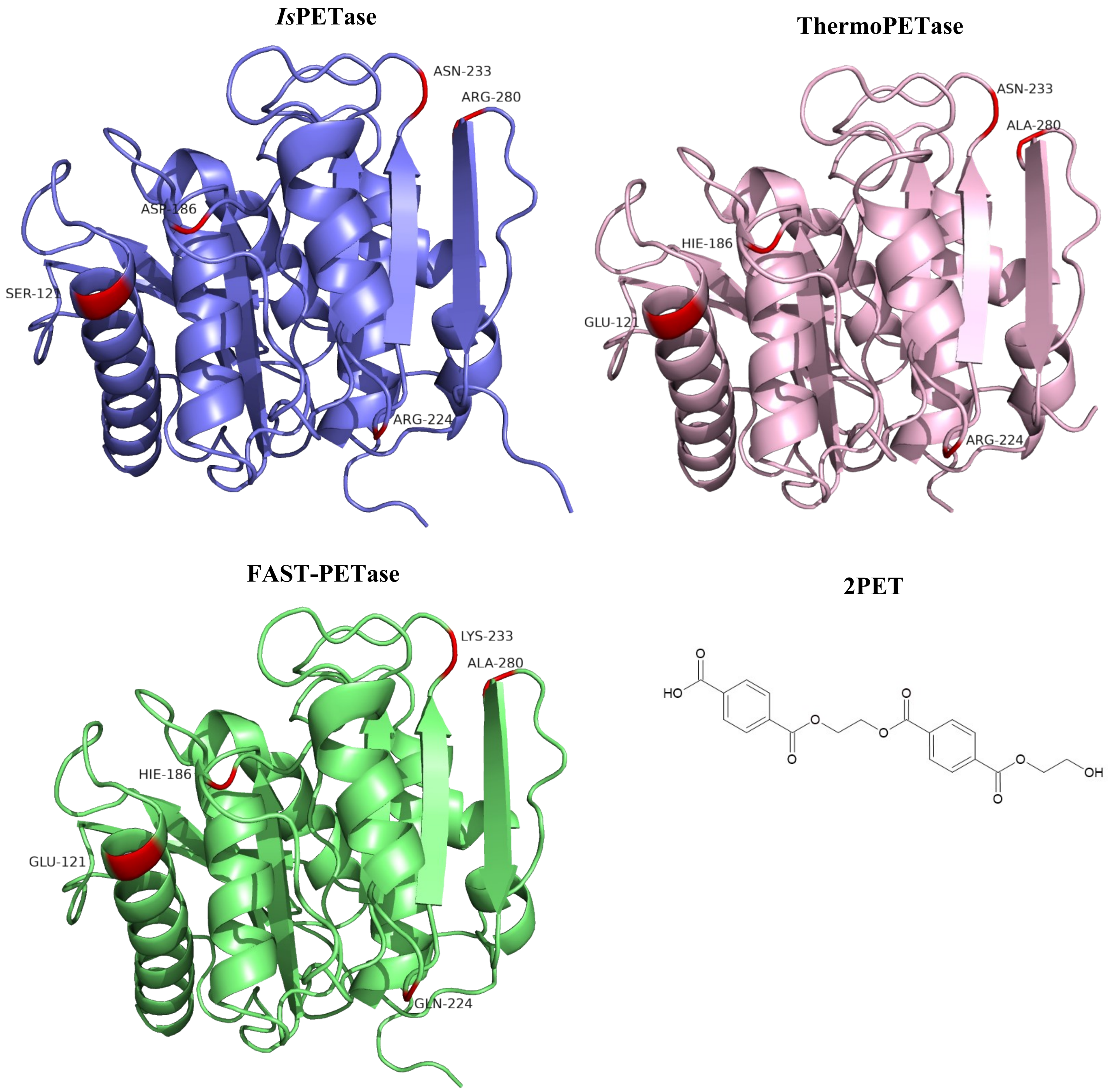

Figure S1. Secondary structures of the proteins: *Is*PETase (PDB ID: 6EQE), ThermoPETase (PDB ID: 6IJ6) and FAST-PETase (PDB ID: 7SH6) and the chemical structure of the dimer PET. Mutations are indicated in red for all three proteins.

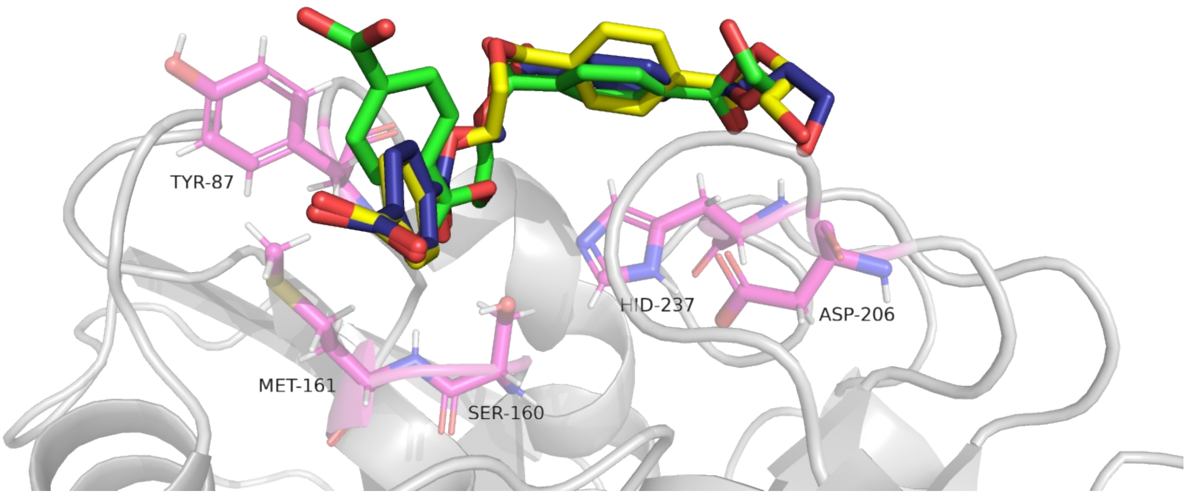

Figure S2. Binding poses (orientations) for the systems *Is*PETase:2PET (yellow), ThermoPETase:2PET (blue) and FAST-PETase (green).

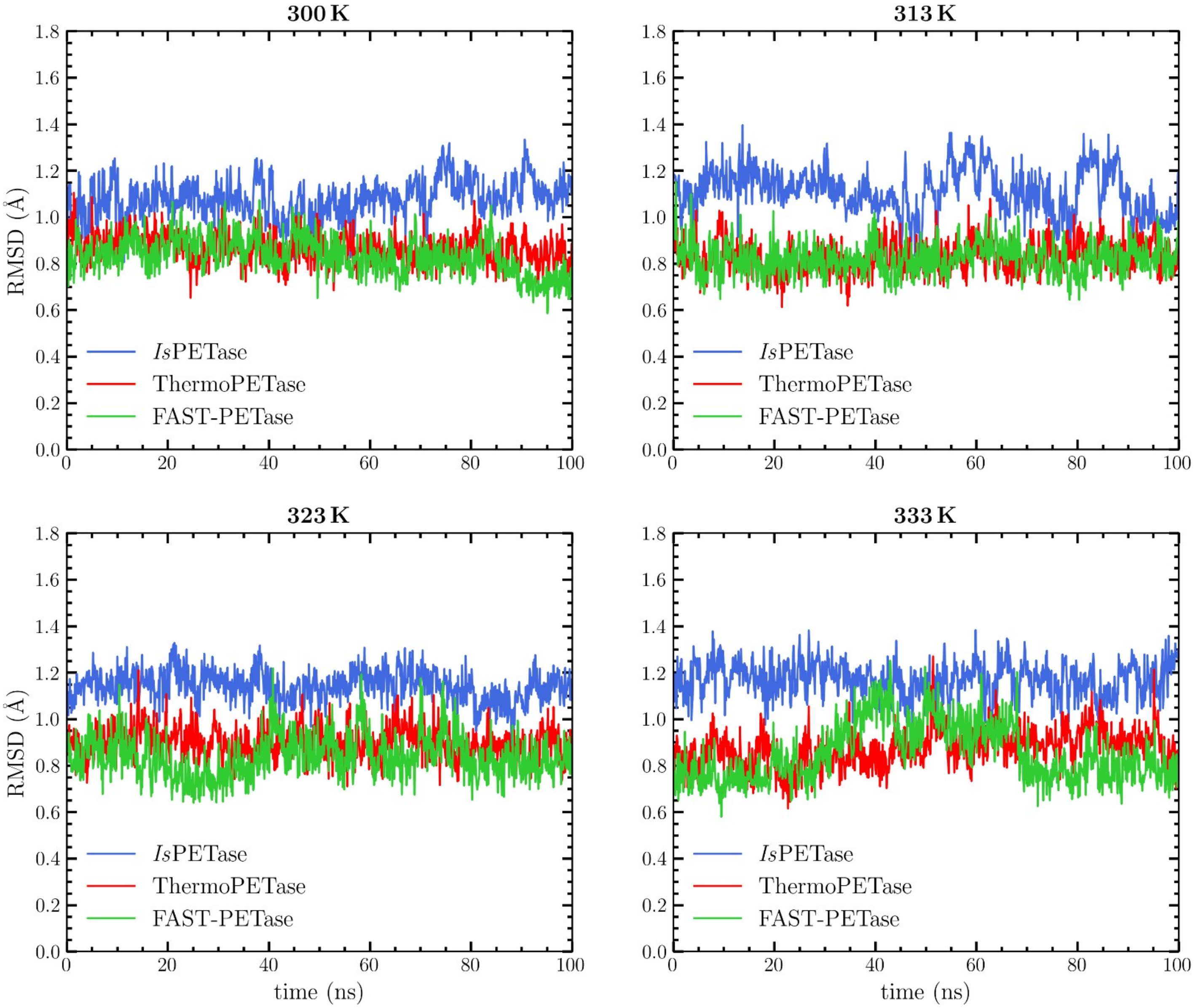

Figure S3. Root Mean Square Deviation (RMSD) of the enzyme backbone atoms for all 12 simulation systems, including *Is*PETase, ThermoPETase, and FAST-PETase at four temperatures (300 K, 313 K, 323 K, and 333 K). RMSD values were computed relative to the initial docked complex.

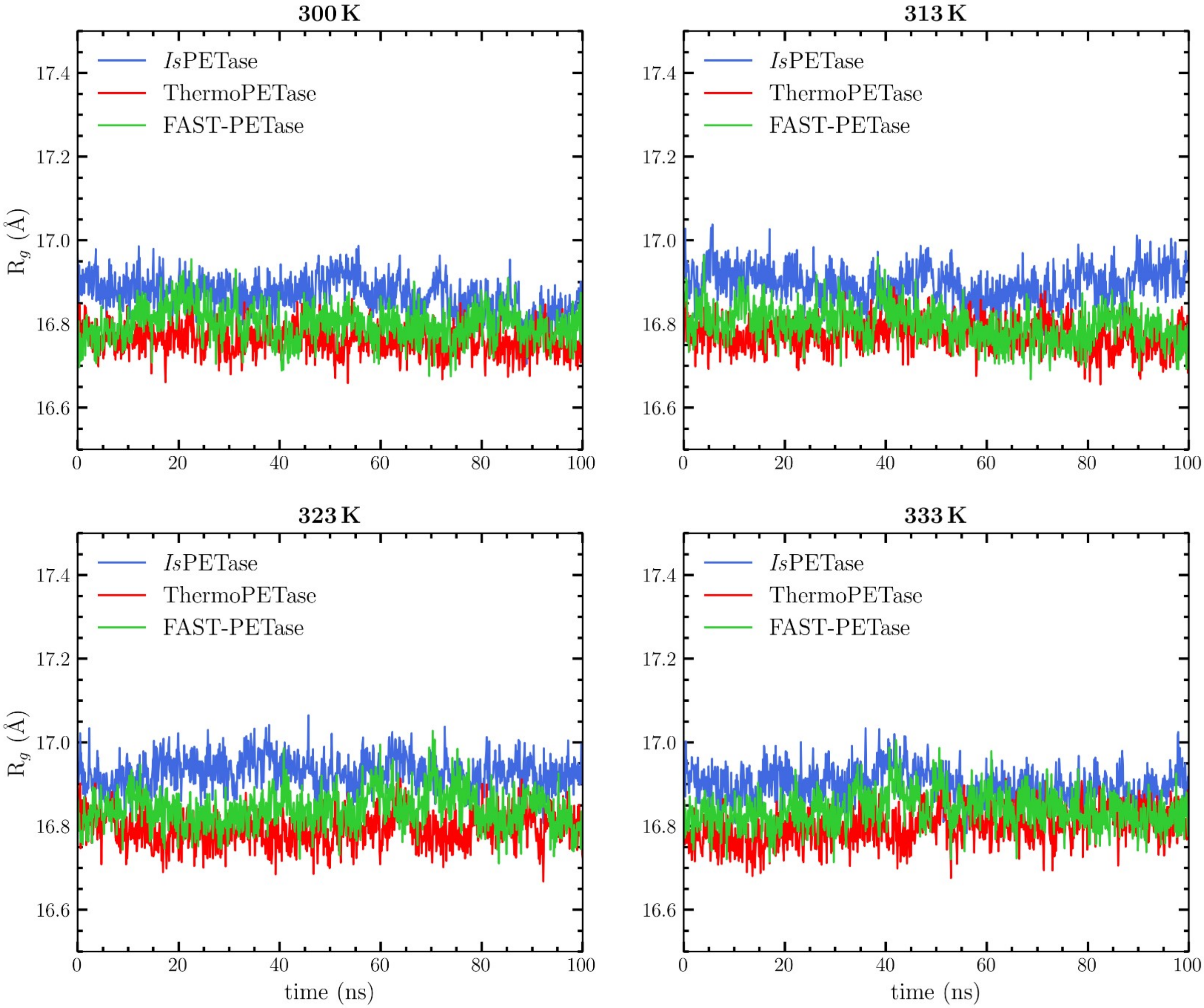

Figure S4. Radius of gyration (R_g) of the enzyme atoms for all 12 simulation systems, including *Is*PETase, ThermoPETase, and FAST-PETase at four temperatures (300 K, 313 K, 323 K, and 333 K). R_g values were calculated throughout the simulations to assess overall structural compactness.

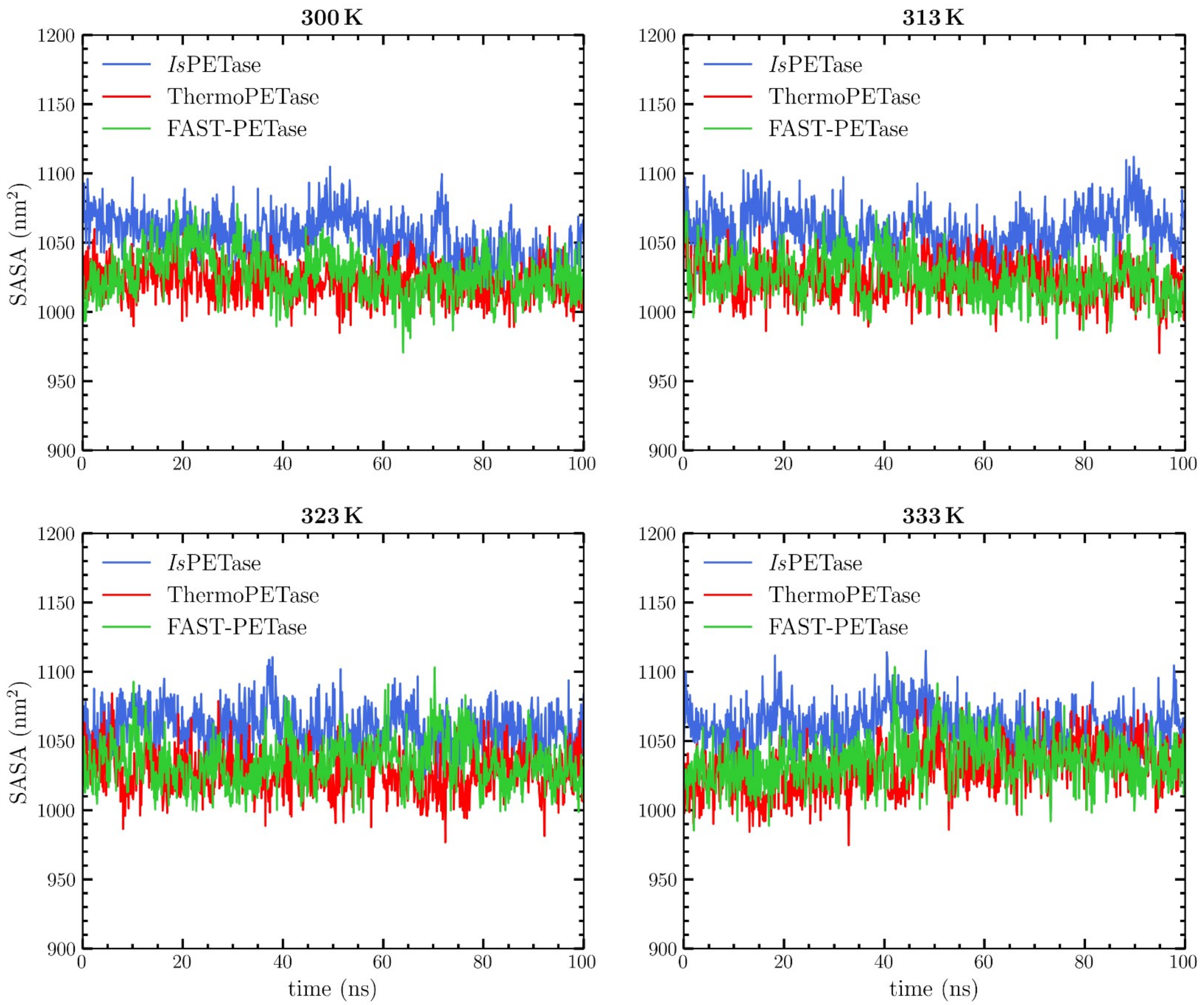

Figure S5. Solvent-accessible surface area (SASA) of the enzyme atoms associated with PET binding for all 12 simulation systems, including *Is*PETase, ThermoPETase, and FAST-PETase at four temperatures (300 K, 313 K, 323 K, and 333 K). SASA values were monitored throughout the simulations to evaluate changes in enzyme surface exposure to the solvent, providing insights into substrate binding.

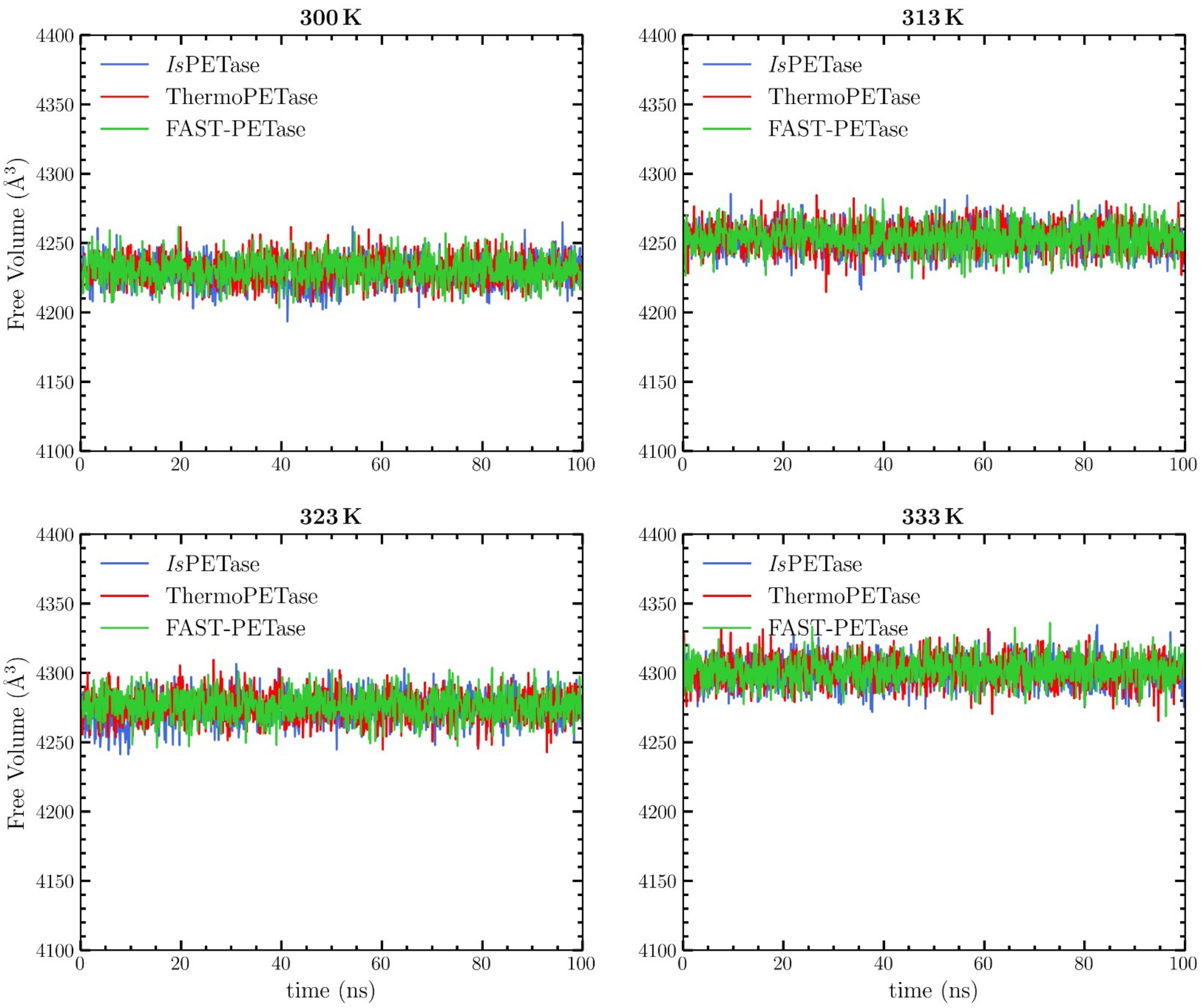

Figure S6. Free volume of the catalytic triad atoms as a function of time for all 12 simulation systems, including *Is*PETase, ThermoPETase, and FAST-PETase, simulated at four temperatures (300, 313, 323, and 333 K). Values were calculated throughout each simulation to assess temperature-dependent fluctuations and flexibility of the catalytic triad in the active site.

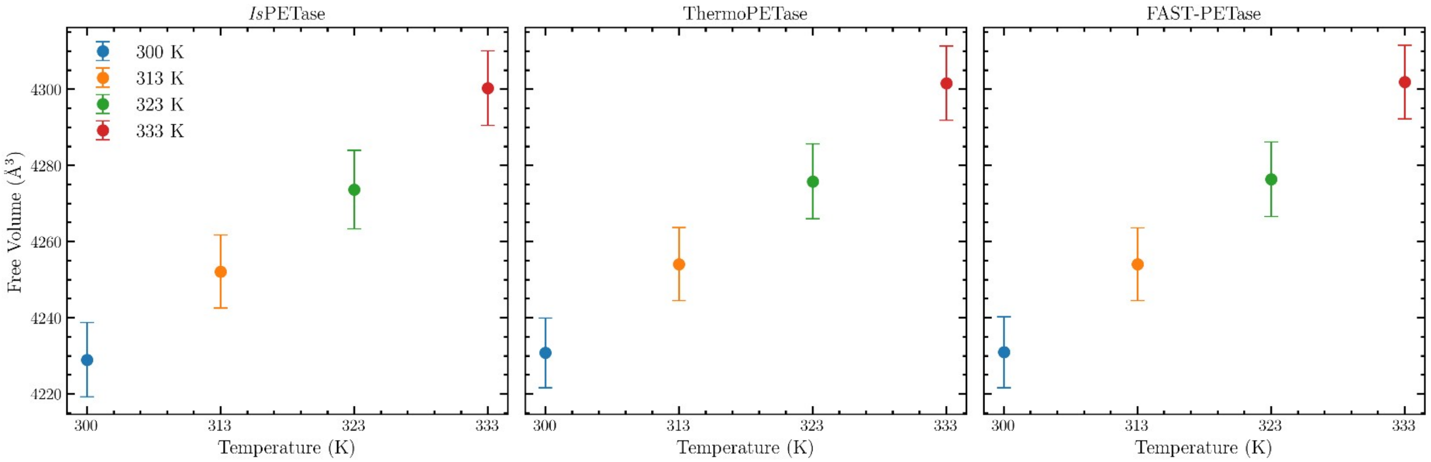

Figure S7. Time-averaged free volume of the catalytic triad atoms as a function of temperature for *Is*PETase, ThermoPETase, and FAST-PETase. Averages were computed over the full simulation trajectories to quantify temperature-dependent changes in active-site flexibility.

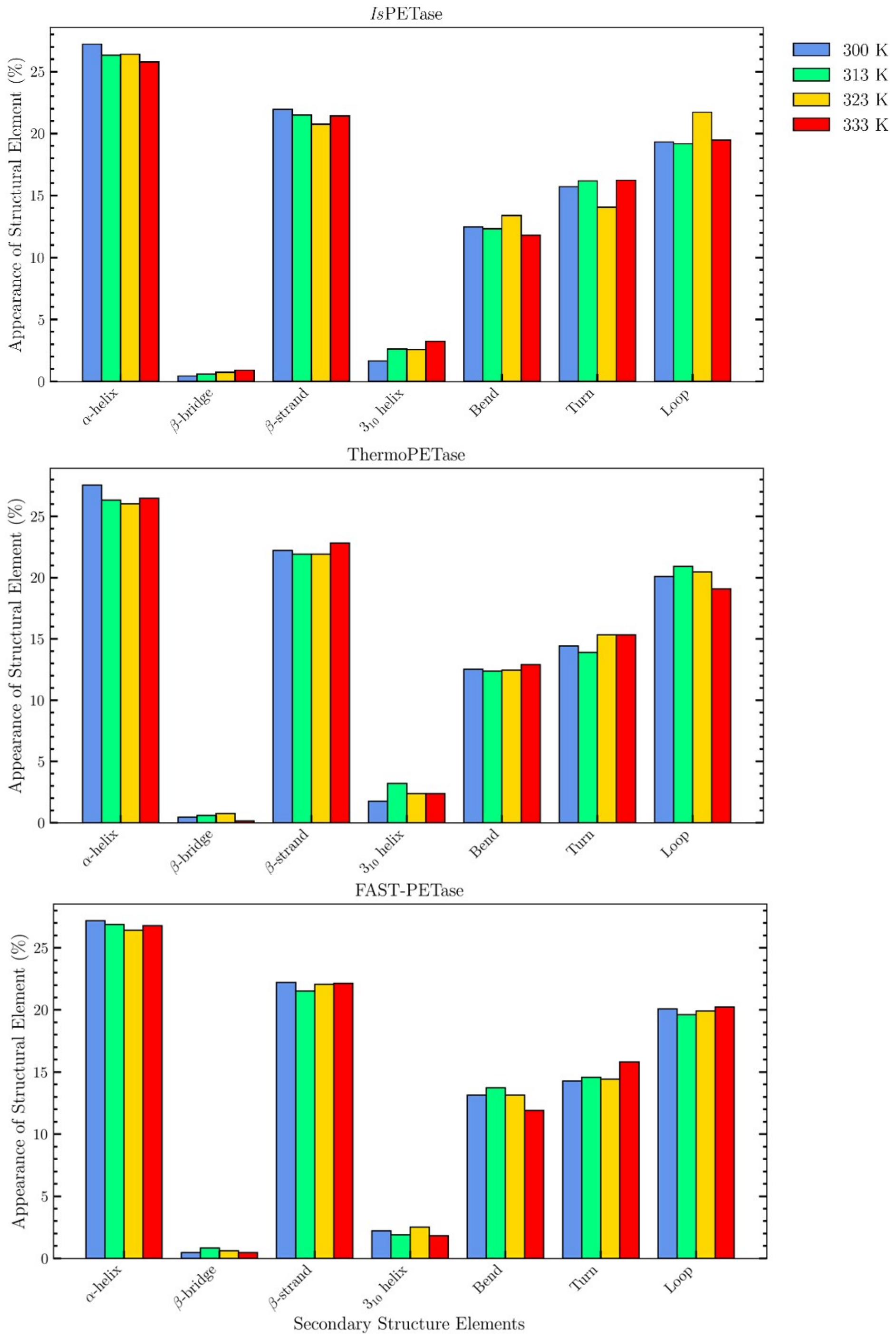

Figure S8. Bar plots illustrating the percentage (%) of appearance of secondary structure elements in the final frame of the simulations. Each subplot corresponds to one enzyme (*Is*PETase, ThermoPETase, and FAST-PETase), comparing results across four temperatures (300 K, 313 K, 323 K, and 333 K).

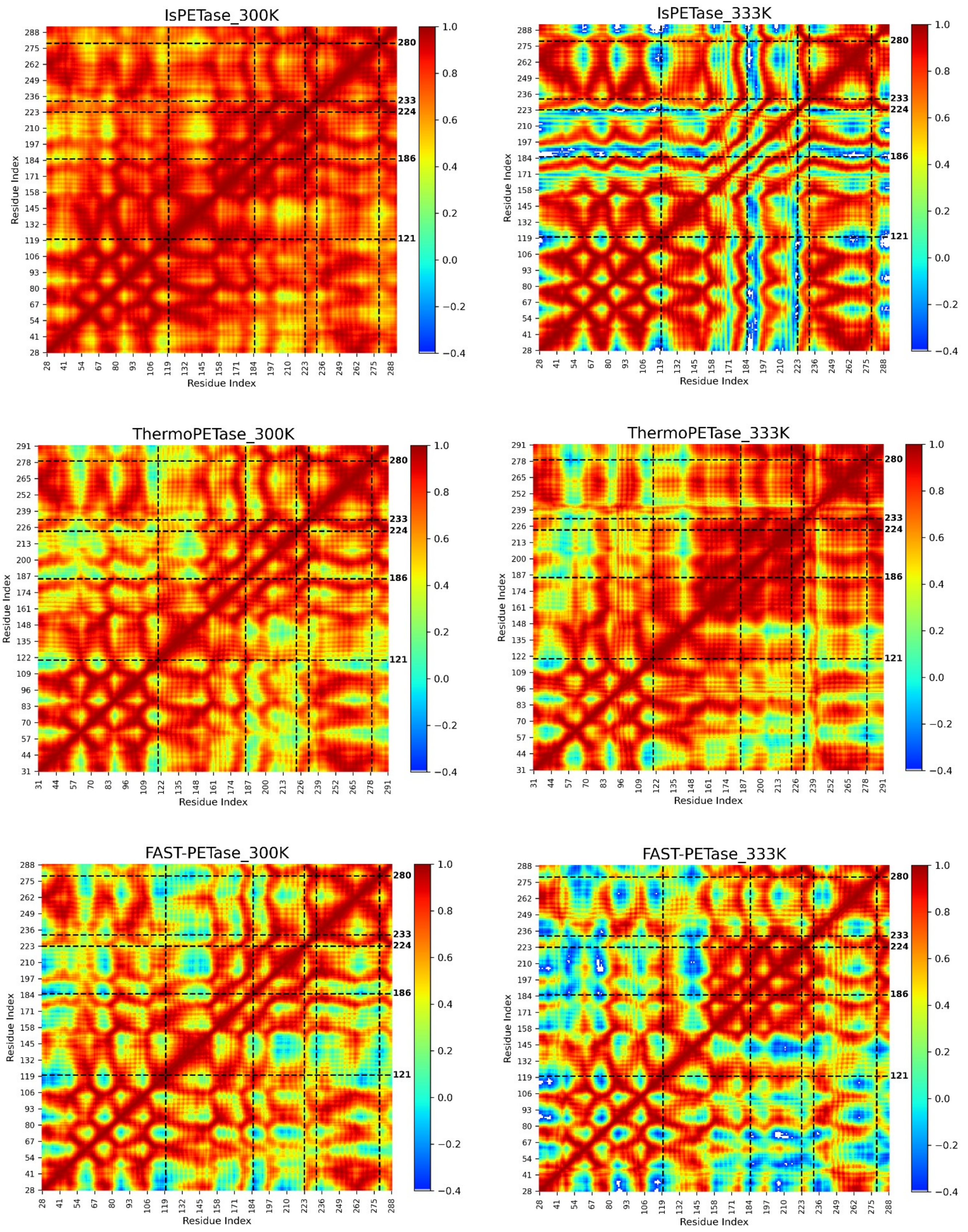

Figure S9. Dynamical cross-correlation matrix (DCCM) analysis of all enzyme-PET complexes at 300 K and 333 K to investigate mutation-induced coupling effects. Residue motions were averaged over the last 10 ns of

each simulation, using the docking conformation as the reference structure. he DCCM was calculated using the MD-TASK tool suite implemented in Python.[84] The color map represents correlation coefficients of residue-residue motions, where red indicates strong positive correlation (1), blue represents anti-correlation (minimum -0.4), and green/cyan corresponds to no correlation. Higher positive correlation reflects more rigid and concerted residue motions, whereas absent correlation indicates more independent and flexible movements. Dashed lines mark the positions of residues mutated in the engineered enzymes.

*Is*PETase

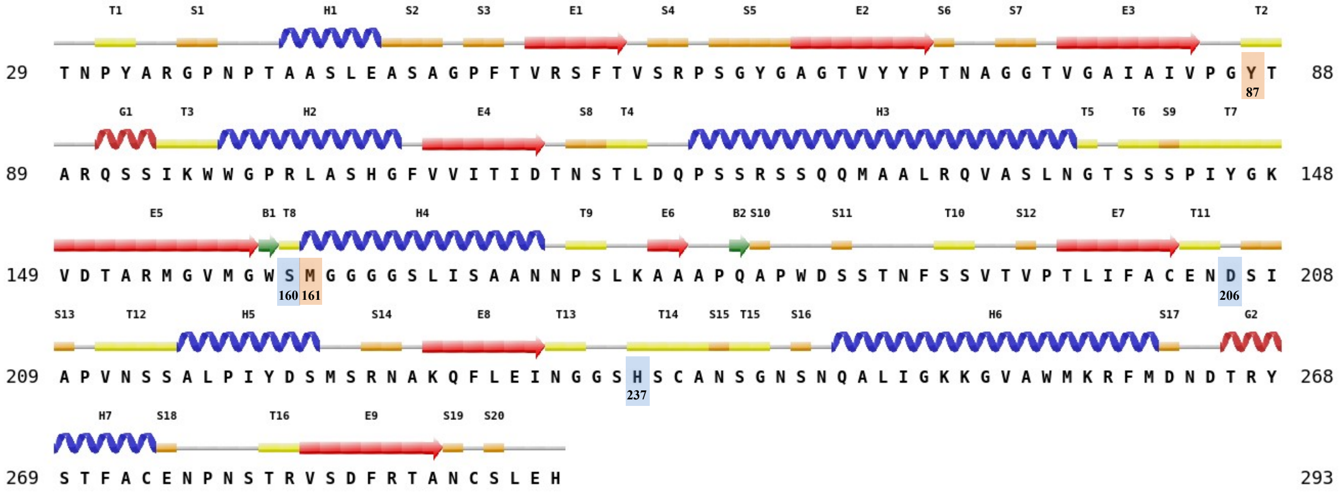

ThermoPETase

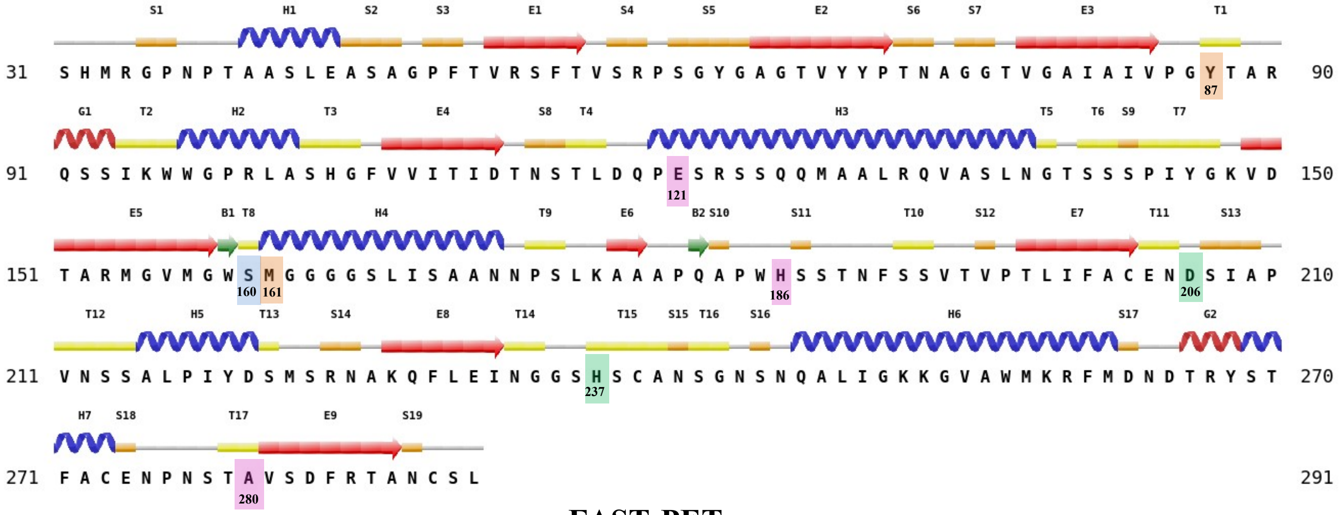

FAST-PETase

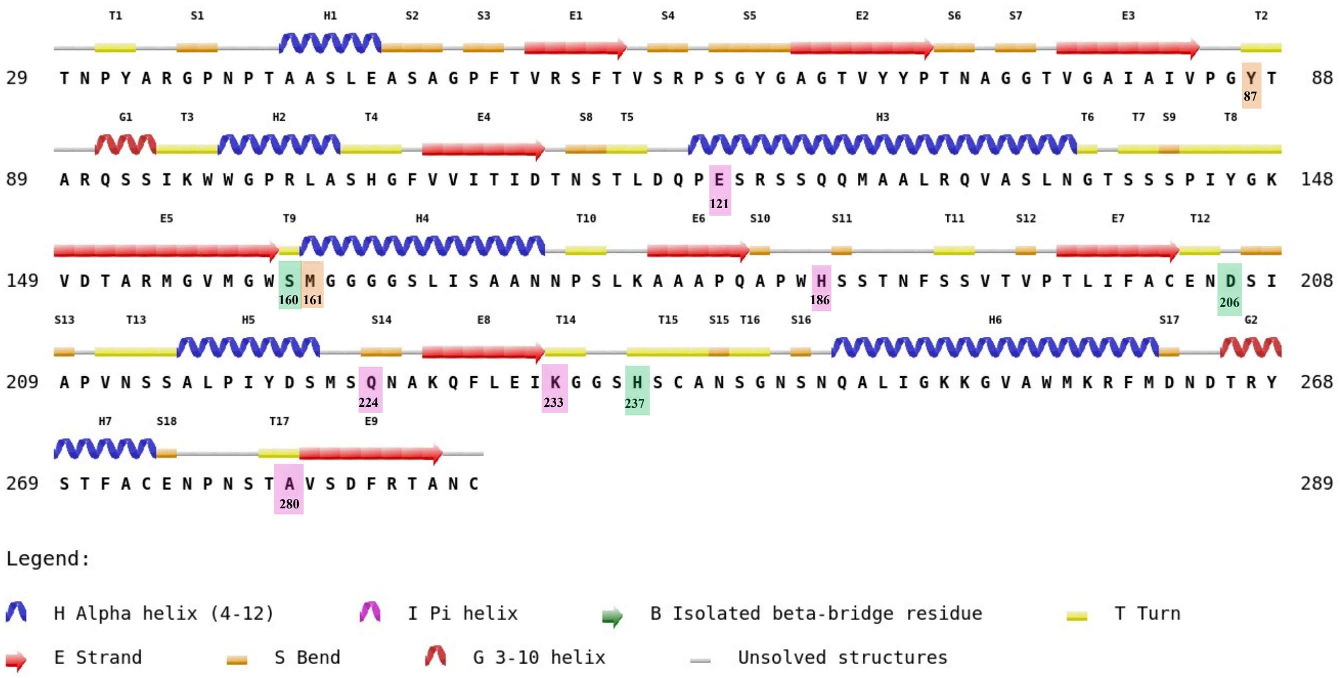

Figure S10. Secondary-structure representation of *Is*PETase, ThermoPETase, and FAST-PETase. Residues are shown as a linear sequence annotated with secondary-structure elements (helices, β-strands, and loops). Catalytic-triad residues (Ser160, His237, Asp206) are highlighted in green, PET-stabilizing residues (Tyr87, Met161) in orange, and engineered mutation (Ser121Glu, Asp186His, Arg224Gln, Asn233Lys and Arg280Ala) sites in pink. The secondary-structure schematics were generated using ProS^{2}Vi, a Python-based visualization tool.[85]

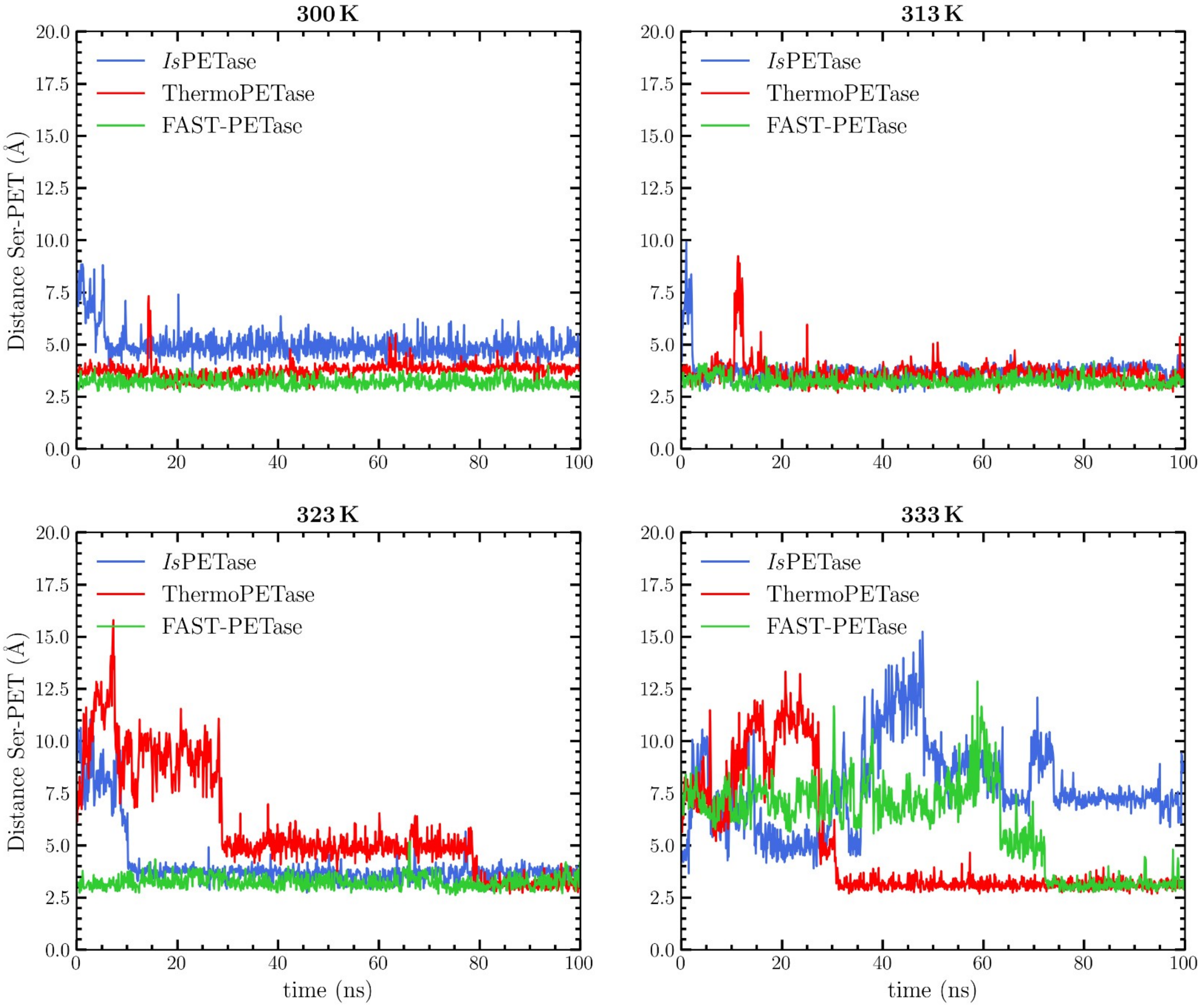

Figure S11. Time evolution of the distance between the hydroxyl oxygen of the catalytic Ser and the ester carbon of PET for all 12 simulation systems (*Is*PETase, ThermoPETase, and FAST-PETase) at 300, 313, 323, and 333 K.

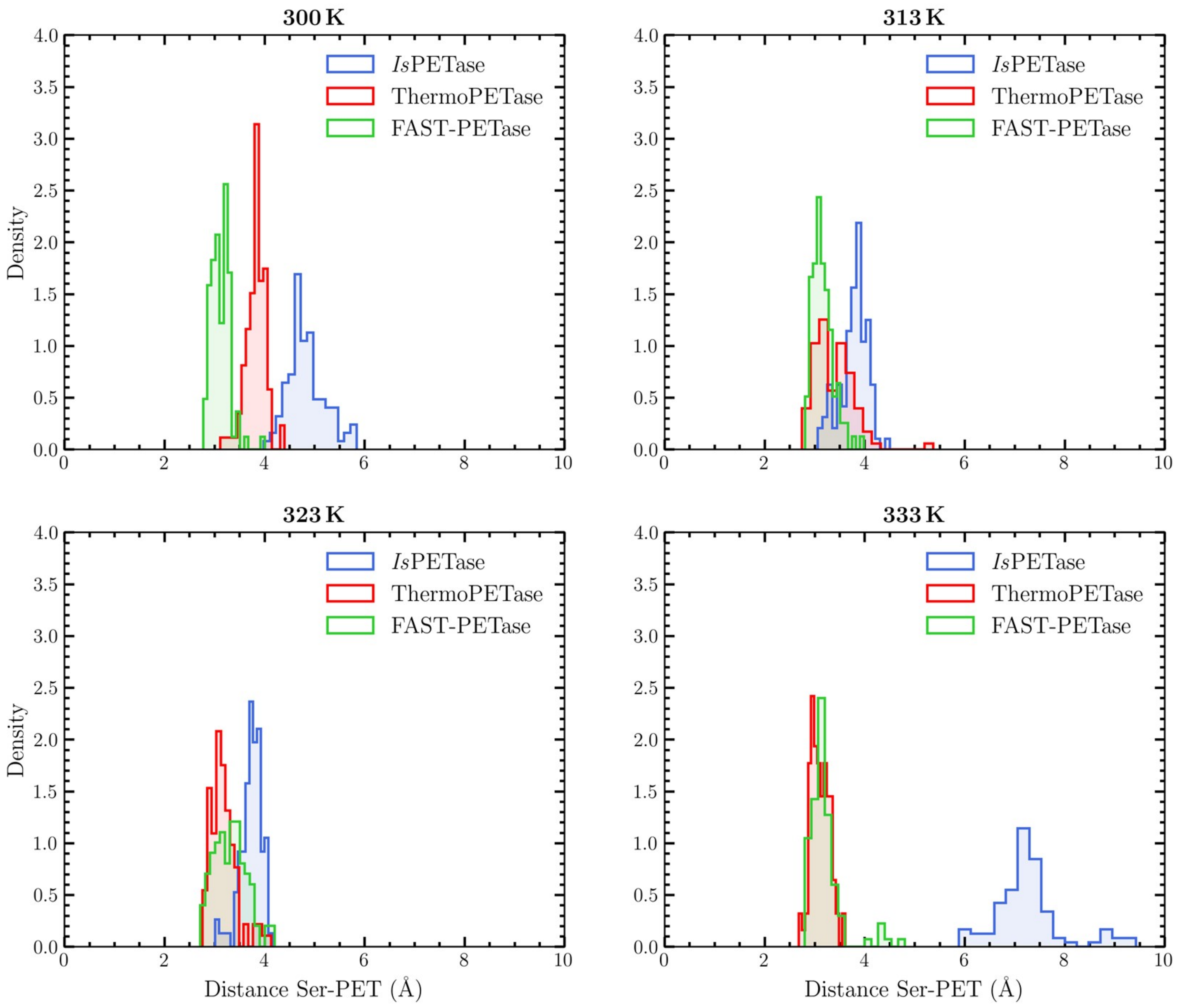

Figure S12. Histogram of the distribution of the distance between the hydroxyl oxygen of the catalytic Ser residue and the ester carbon of PET for all 12 simulation systems, including *Is*PETase, ThermoPETase, and FAST-PETase, simulated at four temperatures (300, 313, 323, and 333 K).

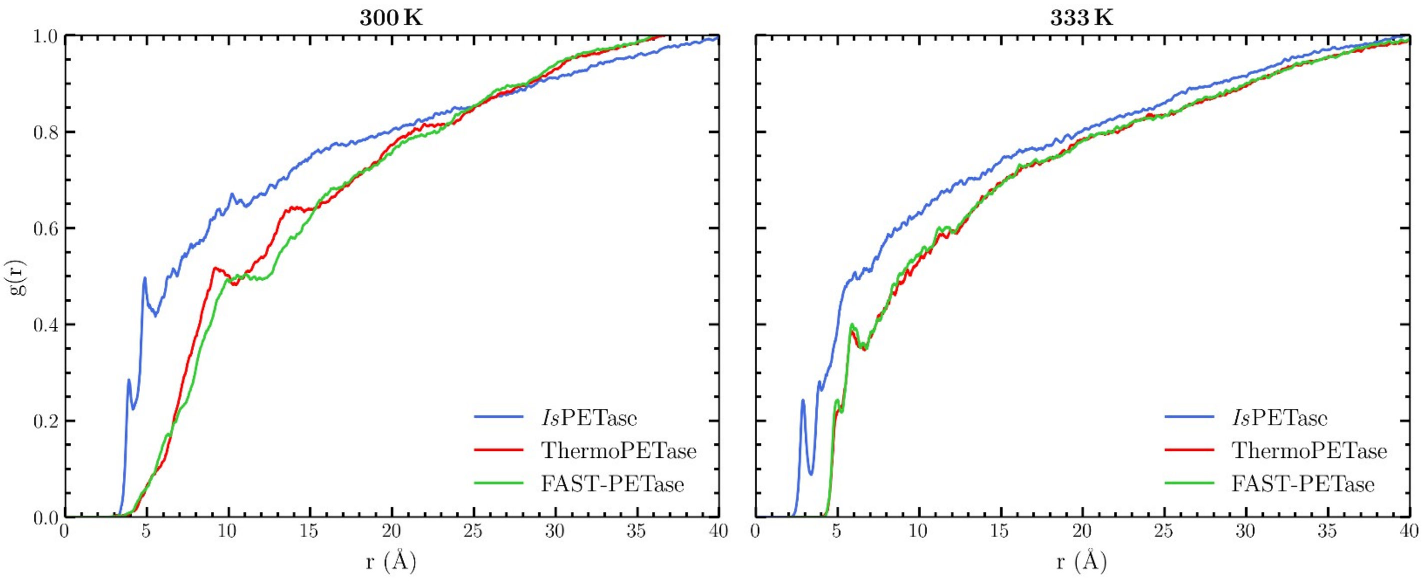

Figure S13. Radial distribution function, *g(r)*, describing the spatial distribution between the ester carbon of PET and the water molecules for all the systems (*Is*PETase, ThermoPETase, and FAST-PETase) at 300 and 333 K. The curves were smoothed using a Savitsky–Golay filter.

REFERENCES

(1) Jerves, C.; Neves, R. P. P.; Ramos, M. J.; da Silva, S.; Fernandes, P. A. Reaction Mechanism of the PET Degrading Enzyme PETase Studied with DFT/MM Molecular Dynamics Simulations. *ACS Catalysis* **2021**, *11* (18), 11626-11638. DOI: 10.1021/acscatal.1c03700.

(2) da Costa, C. H. S.; Dos Santos, A. M.; Alves, C. N.; Marti, S.; Moliner, V.; Santana, K.; Lameira, J. Assessment of the PETase conformational changes induced by poly(ethylene terephthalate) binding. *Proteins* **2021**, *89* (10), 1340-1352. DOI: 10.1002/prot.26155 From NLM Medline.

(3) Boneta, S.; Arafet, K.; Moliner, V. QM/MM Study of the Enzymatic Biodegradation Mechanism of Polyethylene Terephthalate. *J Chem Inf Model* **2021**, *61* (6), 3041-3051. DOI: 10.1021/acs.jcim.1c00394 From NLM Medline.

(4) Feng, S.; Yue, Y.; Zheng, M.; Li, Y.; Zhang, Q.; Wang, W. Is PETase-and Is MHETase-catalyzed cascade degradation mechanism toward polyethylene terephthalate. *ACS Sustainable Chemistry & Engineering* **2021**, *9* (29), 9823-9832. DOI: 10.1021/acssuschemeng.1c02420.

(5) Pinto, A. V.; Ferreira, P.; Neves, R. P.; Fernandes, P. A.; Ramos, M. J.; Magalhaes, A. L. Reaction mechanism of MHETase, a PET degrading enzyme. *ACS Catalysis* **2021**, *11* (16), 10416-10428. DOI: 10.1021/acscatal.1c02444.

(6) Aboelnga, M. M.; Kalyaanamoorthy, S. QM/MM investigation to identify the hallmarks of superior PET biodegradation activity of PETase over cutinase. *ACS Sustainable Chemistry & Engineering* **2022**, *10* (48), 15857-15868. DOI: 10.1021/acssuschemeng.2c04913.

(7) Charupanit, K.; Tipmanee, V.; Sutthibutpong, T.; Limsakul, P. In silico identification of potential sites for a plastic-degrading enzyme by a reverse screening through the protein sequence space and molecular dynamics simulations. *Molecules* **2022**, *27* (10), 3353. DOI: 10.3390/molecules27103353.

(8) James, A.; De, S. Cation–π and hydrophobic interaction controlled PET recognition in double mutated cutinase–identification of a novel binding subsite for better catalytic activity. *RSC advances* **2022**, *12* (32), 20563-20577. DOI: 10.1039/d2ra03394a.

(9) Shrimpton-Phoenix, E.; Mitchell, J. B.; Bühl, M. Computational Insights into the Catalytic Mechanism of Is-PETase: An Enzyme Capable of Degrading Poly (ethylene) Terephthalate. *Chemistry–A European Journal* **2022**, *28* (70), e202201728. DOI: 10.1002/chem.202201728.

(10) Zara, Z.; Mishra, D.; Pandey, S. K.; Csefalvay, E.; Fadaei, F.; Minofar, B.; Reha, D. Surface Interaction of Ionic Liquids: Stabilization of Polyethylene Terephthalate-Degrading Enzymes in Solution. *Molecules* **2021**, *27* (1), 119. DOI: 10.3390/molecules27010119 From NLM Medline.

(11) García-Meseguer, R.; Ortí, E.; Tuñón, I.; Ruiz-Pernía, J. J.; Aragó, J. Insights into the enhancement of the poly (ethylene terephthalate) degradation by FAST-PETase from computational modeling. *Journal of the American Chemical Society* **2023**, *145* (35), 19243-19255. DOI: 10.1021/jacs.3c04427.

(12) Orlando, C.; Prejano, M.; Russo, N.; Marino, T. On the Role of Temperature in the Depolymerization of PET by FAST-PETase: An Atomistic Point of View on Possible Active Site Pre-Organization and Substrate-Destabilization Effects. *Chembiochem* **2023**, *24* (20), e202300412. DOI: 10.1002/cbic.202300412 From NLM Medline.

(13) Braga, B.; Silva, M.; Amaral, M. Variations of the canonical triad of IsPETase: In silico insights with molecular dynamics simulation. *Chinese Journal of Physics* **2023**, *84*, 282-291. DOI: 10.1016/j.cjph.2023.05.022.

(14) Nelson, I.; Ramos, R. Computational assessment on catalytic activity of PET hydrolase. *bioRxiv* **2023**, 2023.2005. 2030.542930. DOI: 10.1101/2023.05.30.542930.

(15) Wang, N.; Li, Y.; Zheng, M.; Dong, W.; Zhang, Q.; Wang, W. BhrPETase catalyzed polyethylene terephthalate depolymerization: A quantum mechanics/molecular mechanics approach. *Journal of Hazardous Materials* **2024**, *477*, 135414. DOI: 10.1016/j.jhazmat.2024.135414.

(16) Berselli, A.; Ramos, M. J.; Menziani, M. C. Novel Pet-Degrading Enzymes: Structure-Function from a Computational Perspective. *Chembiochem* **2021**, *22* (12), 2032-2050. DOI: 10.1002/cbic.202000841 From NLM Medline.

(17) Burgin, T.; Pollard, B. C.; Knott, B. C.; Mayes, H. B.; Crowley, M. F.; McGeehan, J. E.; Beckham, G. T.; Woodcock, H. L. The reaction mechanism of the Ideonella sakaiensis PETase enzyme. *Commun Chem* **2024**, *7* (1), 65. DOI: 10.1038/s42004-024-01154-x From NLM PubMed-not-MEDLINE.

(18) Jerves, C.; Neves, R. P.; da Silva, S. L.; Ramos, M. J.; Fernandes, P. A. Rate-enhancing PETase mutations determined through DFT/MM molecular dynamics simulations. *New Journal of Chemistry* **2024**, *48* (1), 45-54. DOI: 10.1039/d3nj04204a.

(19) Pinto, E. S. M.; Mangini, A. T.; Novo, L. C. C.; Cavatao, F. G.; Krause, M. J.; Dorn, M. Assessment of Kaistella jeonii esterase conformational dynamics in response to poly (ethylene terephthalate) binding. *Current Research in Structural Biology* **2024**, *7*, 100130.

(20) Dos Santos, A. M.; da Costa, C. H.; Silva, P. H.; Skaf, M. S.; Lameira, J. Exploring the Reaction Mechanism of Polyethylene Terephthalate Biodegradation through QM/MM Approach. *The Journal of Physical Chemistry B* **2024**. DOI: 10.1021/acs.jpcb.4c02207.

(21) Zheng, M.; Li, Y.; Dong, W.; Zhang, Q.; Wang, W. Hydrolase-Catalyzed Depolymerization Mechanism toward Crystalline and Amorphous Polyethylene Terephthalate. *ACS Sustainable Chemistry & Engineering* **2024**, *12* (27), 10252-10259. DOI: 10.1021/acssuschemeng.4c02986.

(22) Jackering, A.; van der Kamp, M.; Strodel, B.; Zinovjev, K. Influence of Wobbling Tryptophan and Mutations on PET Degradation Explored by QM/MM Free Energy Calculations. *J Chem Inf Model* **2024**, *64* (19), 7544-7554. DOI: 10.1021/acs.jcim.4c00776 From NLM Medline.

(23) Xu, S.; Huo, C.; Chu, X. Unraveling the Interplay between Stability and Flexibility in the Design of Polyethylene Terephthalate (PET) Hydrolases. *Journal of Chemical Information and Modeling* **2024**, *64* (19), 7576-7589. DOI: 10.1021/acs.jcim.4c00877.

(24) James, A.; Bhasi, A.; De, S. Bridging the Gap in the Structure-Function Paradigm of Enzymatic PET Degradation-Aromatic Residue Driven Balanced Interactions with Catalytic and Anchoring Subsite. *Chembiochem* **2024**, *25* (21), e202400555. DOI: 10.1002/cbic.202400555 From NLM Medline.

(25) Sahihi, M.; Fayon, P.; Nauton, L.; Goujon, F.; Devemy, J.; Dequidt, A.; Hauret, P.; Malfreyt, P. Probing Enzymatic PET Degradation: Molecular Dynamics Analysis of Cutinase Adsorption and Stability. *J Chem Inf Model* **2024**, *64* (10), 4112-4120. DOI: 10.1021/acs.jcim.4c00079 From NLM Medline.

(26) de Oliveira, F. K.; Santos, L. O.; Buffon, J. G. Mechanism of action, sources, and application of peroxidases. *Food Res Int* **2021**, *143*, 110266. DOI: 10.1016/j.foodres.2021.110266 From NLM Medline.

(27) Wei, R.; Oeser, T.; Then, J.; Kuhn, N.; Barth, M.; Schmidt, J.; Zimmermann, W. Functional characterization and structural modeling of synthetic polyester-degrading hydrolases from Thermomonospora curvata. *AMB Express* **2014**, *4*, 44. DOI: 10.1186/s13568-014-0044-9 From NLM PubMed-not-MEDLINE.

(28) Then, J.; Wei, R.; Oeser, T.; Barth, M.; Belisario-Ferrari, M. R.; Schmidt, J.; Zimmermann, W. Ca2+ and Mg2+ binding site engineering increases the degradation of polyethylene terephthalate films by polyester hydrolases from Thermobifida fusca. *Biotechnol J* **2015**, *10* (4), 592-598. DOI: 10.1002/biot.201400620 From NLM Medline.

(29) Fecker, T.; Galaz-Davison, P.; Engelberger, F.; Narui, Y.; Sotomayor, M.; Parra, L. P.; Ramirez-Sarmiento, C. A. Active Site Flexibility as a Hallmark for Efficient PET Degradation by I. sakaiensis PETase. *Biophys J* **2018**, *114* (6), 1302-1312. DOI: 10.1016/j.bpj.2018.02.005 From NLM Medline.

(30) Austin, H. P.; Allen, M. D.; Donohoe, B. S.; Rorrer, N. A.; Kearns, F. L.; Silveira, R. L.; Pollard, B. C.; Dominick, G.; Duman, R.; El Omari, K.; et al. Characterization and engineering of a plastic-degrading aromatic polyesterase. *Proc Natl Acad Sci U S A* **2018**, *115* (19), E4350-E4357. DOI: 10.1073/pnas.1718804115 From NLM Medline.

(31) Knott, B. C.; Erickson, E.; Allen, M. D.; Gado, J. E.; Graham, R.; Kearns, F. L.; Pardo, I.; Topuzlu, E.; Anderson, J. J.; Austin, H. P.; et al. Characterization and engineering of a two-enzyme system for plastics depolymerization. *Proc Natl Acad Sci U S A* **2020**, *117* (41), 25476-25485. DOI: 10.1073/pnas.2006753117 From NLM Medline.

(32) Tournier, V.; Topham, C. M.; Gilles, A.; David, B.; Folgoas, C.; Moya-Leclair, E.; Kamionka, E.; Desrousseaux, M. L.; Texier, H.; Gavalda, S.; et al. An engineered PET depolymerase to break down and recycle plastic bottles. *Nature* **2020**, *580* (7802), 216-219. DOI: 10.1038/s41586-020-2149-4 From NLM Medline.

(33) Zheng, M.; Li, Y.; Dong, W.; Feng, S.; Zhang, Q.; Wang, W. Computational biotransformation of polyethylene terephthalate by depolymerase: A QM/MM approach. *J Hazard Mater* **2022**, *423* (Pt A), 127017. DOI: 10.1016/j.jhazmat.2021.127017 From NLM Medline.

(34) Meng, X.; Yang, L.; Liu, H.; Li, Q.; Xu, G.; Zhang, Y.; Guan, F.; Zhang, Y.; Zhang, W.; Wu, N.; Tian, J. Protein engineering of stable IsPETase for PET plastic degradation by Premuse. *Int J Biol Macromol* **2021**, *180*, 667-676. DOI: 10.1016/j.ijbiomac.2021.03.058 From NLM Medline.

(35) Liu, Y.; Liu, Z.; Guo, Z.; Yan, T.; Jin, C.; Wu, J. Enhancement of the degradation capacity of IsPETase for PET plastic degradation by protein engineering. *Sci Total Environ* **2022**, *834*, 154947. DOI: 10.1016/j.scitotenv.2022.154947 From NLM Medline.

(36) Weigert, S.; Perez-Garcia, P.; Gisdon, F. J.; Gagsteiger, A.; Schweinshaut, K.; Ullmann, G. M.; Chow, J.; Streit, W. R.; Hocker, B. Investigation of the halophilic PET hydrolase PET6 from Vibrio gazogenes. *Protein Sci* **2022**, *31* (12), e4500. DOI: 10.1002/pro.4500 From NLM Medline.

(37) Chen, X. Q.; Guo, Z. Y.; Wang, L.; Yan, Z. F.; Jin, C. X.; Huang, Q. S.; Kong, D. M.; Rao, D. M.; Wu, J. Directional-path modification strategy enhances PET hydrolase catalysis of plastic degradation. *J Hazard Mater* **2022**, *433*, 128816. DOI: 10.1016/j.jhazmat.2022.128816 From NLM Medline.

(38) Yin, Q.; You, S.; Zhang, J.; Qi, W.; Su, R. Enhancement of the polyethylene terephthalate and mono-(2-hydroxyethyl) terephthalate degradation activity of Ideonella sakaiensis PETase by an electrostatic interaction-based strategy. *Bioresour Technol* **2022**, *364*, 128026. DOI: 10.1016/j.biortech.2022.128026 From NLM PubMed-not-MEDLINE.

(39) Pirillo, V.; Orlando, M.; Tessaro, D.; Pollegioni, L.; Molla, G. An Efficient Protein Evolution Workflow for the Improvement of Bacterial PET Hydrolyzing Enzymes. *Int J Mol Sci* **2021**, *23* (1), 264. DOI: 10.3390/ijms23010264 From NLM Medline.

(40) Mrigwani, A.; Pitaliya, M.; Kaur, H.; Kasilingam, B.; Thakur, B.; Guptasarma, P. Rational mutagenesis of Thermobifida fusca cutinase to modulate the enzymatic degradation of polyethylene terephthalate. *Biotechnol Bioeng* **2023**, *120* (3), 674-686. DOI: 10.1002/bit.28305 From NLM Medline.

(41) von Haugwitz, G.; Han, X.; Pfaff, L.; Li, Q.; Wei, H.; Gao, J.; Methling, K.; Ao, Y.; Brack, Y.; Mican, J. Structural insights into (Tere) phthalate-Ester hydrolysis by a carboxylesterase and its role in promoting PET depolymerization. ACS Catal. 12, 15259–15270. 2022.

(42) Guo, B.; Vanga, S. R.; Lopez-Lorenzo, X.; Saenz-Mendez, P.; Ericsson, S. R.; Fang, Y.; Ye, X.; Schriever, K.; Backstrom, E.; Biundo, A. Conformational selection in biocatalytic plastic degradation by PETase. *Acs Catalysis* **2022**, *12* (6), 3397-3409. DOI: 10.1021/acscatal.1c05548.

(43) Aristizábal-Lanza, L.; Mankar, S. V.; Tullberg, C.; Zhang, B.; Linares-Pastén, J. A. Comparison of the enzymatic depolymerization of polyethylene terephthalate and AkestraTM using Humicola insolens cutinase. *Frontiers in Chemical Engineering* **2022**, *4*, 1048744. DOI: 10.3389/fceng.2022.1048744.

(44) Pfaff, L.; Gao, J.; Li, Z.; Jackering, A.; Weber, G.; Mican, J.; Chen, Y.; Dong, W.; Han, X.; Feiler, C. G.; et al. Multiple Substrate Binding Mode-Guided Engineering of a Thermophilic PET Hydrolase. *ACS Catal* **2022**, *12* (15), 9790-9800. DOI: 10.1021/acscatal.2c02275 From NLM PubMed-not-MEDLINE.

(45) Waltmann, C.; Mills, C. E.; Wang, J.; Qiao, B.; Torkelson, J. M.; Tullman-Ercek, D.; Olvera de la Cruz, M. Functional enzyme-polymer complexes. *Proc Natl Acad Sci U S A* **2022**, *119* (13), e2119509119. DOI: 10.1073/pnas.2119509119 From NLM Medline.

(46) Liu, Y.; Liu, C.; Liu, H.; Zeng, Q.; Tian, X.; Long, L.; Yang, J. Catalytic Features and Thermal Adaptation Mechanisms of a Deep Sea Bacterial Cutinase-Type Poly(Ethylene Terephthalate) Hydrolase. *Front Bioeng Biotechnol* **2022**, *10*, 865787. DOI: 10.3389/fbioe.2022.865787 From NLM PubMed-not-MEDLINE.

(47) Sevilla, M. E.; Garcia, M. D.; Perez-Castillo, Y.; Armijos-Jaramillo, V.; Casado, S.; Vizuete, K.; Debut, A.; Cerda-Mejia, L. Degradation of PET Bottles by an Engineered Ideonella sakaiensis PETase. *Polymers (Basel)* **2023**, *15* (7), 1779. DOI: 10.3390/polym15071779 From NLM PubMed-not-MEDLINE.

(48) Crnjar, A.; Griñen, A.; Kamerlin, S. C.; Ramírez-Sarmiento, C. A. Conformational selection of a tryptophan side chain drives the generalized increase in activity of PET hydrolases through a Ser/Ile double mutation. *ACS organic & inorganic Au* **2023**, *3* (2), 109-119. DOI: 10.1021/acsorginorgau.2c00054.

(49) Qu, Z.; Chen, K.; Zhang, L.; Sun, Y. Computation-Based Design of Salt Bridges in PETase for Enhanced Thermostability and Performance for PET Degradation. *Chembiochem* **2023**, *24* (21), e202300373. DOI: 10.1002/cbic.202300373 From NLM Medline.

(50) Meng, S.; Li, Z.; Zhang, P.; Contreras, F.; Ji, Y.; Schwaneberg, U. Deep learning guided enzyme engineering of Thermobifida fusca cutinase for increased PET depolymerization. *Chinese Journal of Catalysis* **2023**, *50*, 229-238. DOI: 10.1016/S1872-2067(23)64470-5.

(51) Ding, Z.; Xu, G.; Miao, R.; Wu, N.; Zhang, W.; Yao, B.; Guan, F.; Huang, H.; Tian, J. Rational redesign of thermophilic PET hydrolase LCCICCG to enhance hydrolysis of high crystallinity polyethylene terephthalates. *J Hazard Mater* **2023**, *453*, 131386. DOI: 10.1016/j.jhazmat.2023.131386 From NLM Medline.

(52) Shi, L.; Liu, P.; Tan, Z.; Zhao, W.; Gao, J.; Gu, Q.; Ma, H.; Liu, H.; Zhu, L. Complete Depolymerization of PET Wastes by an Evolved PET Hydrolase from Directed Evolution. *Angew Chem Int Ed Engl* **2023**, *62* (14), e202218390. DOI: 10.1002/anie.202218390 From NLM Medline.

(53) Swiderek, K.; Velasco-Lozano, S.; Galmes, M. A.; Olazabal, I.; Sardon, H.; Lopez-Gallego, F.; Moliner, V. Mechanistic studies of a lipase unveil effect of pH on hydrolysis products of small PET modules. *Nat Commun* **2023**, *14* (1), 3556. DOI: 10.1038/s41467-023-39201-1 From NLM Medline.

(54) Pirillo, V.; Orlando, M.; Battaglia, C.; Pollegioni, L.; Molla, G. Efficient polyethylene terephthalate degradation at moderate temperature: a protein engineering study of LC-cutinase highlights the key role of residue 243. *The FEBS journal* **2023**, *290* (12), 3185-3202. DOI: 10.1111/febs.16736.

(55) Zhang, J.; Wang, H.; Luo, Z.; Yang, Z.; Zhang, Z.; Wang, P.; Li, M.; Zhang, Y.; Feng, Y.; Lu, D.; Zhu, Y. Computational design of highly efficient thermostable MHET hydrolases and dual enzyme system for PET recycling. *Commun Biol* **2023**, *6* (1), 1135. DOI: 10.1038/s42003-023-05523-5 From NLM Medline.

(56) Richter, P. K.; Blazquez-Sanchez, P.; Zhao, Z.; Engelberger, F.; Wiebeler, C.; Kunze, G.; Frank, R.; Krinke, D.; Frezzotti, E.; Lihanova, Y.; et al. Structure and function of the metagenomic plastic-degrading polyester hydrolase PHL7 bound to its product. *Nat Commun* **2023**, *14* (1), 1905. DOI: 10.1038/s41467-023-37415-x From NLM Medline.

(57) Falkenstein, P.; Zhao, Z.; Di Pede-Mattatelli, A.; Künze, G.; Sommer, M.; Sonnendecker, C.; Zimmermann, W.; Colizzi, F.; Matysik, J. r.; Song, C. On the binding mode and molecular mechanism of enzymatic polyethylene terephthalate degradation. *ACS Catalysis* **2023**, *13* (10), 6919-6933. DOI: 10.1021/acscatal.3c00259.

(58) Li, A.; Sheng, Y.; Cui, H.; Wang, M.; Wu, L.; Song, Y.; Yang, R.; Li, X.; Huang, H. Discovery and mechanism-guided engineering of BHET hydrolases for improved PET recycling and upcycling. *Nat Commun* **2023**, *14* (1), 4169. DOI: 10.1038/s41467-023-39929-w From NLM Medline.

(59) Lee, S. H.; Seo, H.; Hong, H.; Park, J.; Ki, D.; Kim, M.; Kim, H. J.; Kim, K. J. Three-directional engineering of IsPETase with enhanced protein yield, activity, and durability. *J Hazard Mater* **2023**, *459*, 132297. DOI: 10.1016/j.jhazmat.2023.132297 From NLM Medline.

(60) Qu, Z.; Zhang, L.; Sun, Y. Molecular Insights into the Enhanced Activity and/or Thermostability of PET Hydrolase by D186 Mutations. *Molecules* **2024**, *29* (6), 1338. DOI: 10.3390/molecules29061338.

(61) Ding, K.; Levitskaya, Z.; Sana, B.; Pasula, R. R.; Kannan, S.; Adam, A.; Sundaravadanam, V. V.; Verma, C.; Lim, S.; Ghadessy, J. F. Modulation of PETase active site flexibility and activity on morphologically distinct polyethylene terephthalate substrates by surface charge engineering. *Biochemical Engineering Journal* **2024**, *209*, 109420. DOI: 10.1016/j.bej.2024.109420.

(62) Cui, Y.; Chen, Y.; Sun, J.; Zhu, T.; Pang, H.; Li, C.; Geng, W.-C.; Wu, B. Computational redesign of a hydrolase for nearly complete PET depolymerization at industrially relevant high-solids loading. *Nature Communications* **2024**, *15* (1), 1417. DOI: 10.1038/s41467-024-45662-9.

(63) Han, Z.; Nina, M. R. H.; Zhang, X.; Huang, H.; Fan, D.; Bai, Y. Discovery and characterization of two novel polyethylene terephthalate hydrolases: One from a bacterium identified in human feces and one from the Streptomyces genus. *J Hazard Mater* **2024**, *472*, 134532. DOI: 10.1016/j.jhazmat.2024.134532 From NLM Medline.

(64) Lu, D.; Chen, Y.; Jin, S.; Wu, Q.; Wu, J.; Liu, J.; Wang, F.; Deng, L.; Nie, K. The evolution of cutinase Est1 based on the clustering strategy and its application for commercial PET bottles degradation. *J Environ Manage* **2024**, *368*, 122217. DOI: 10.1016/j.jenvman.2024.122217 From NLM Medline.

(65) Joho, Y.; Royan, S.; Caputo, A. T.; Newton, S.; Peat, T. S.; Newman, J.; Jackson, C.; Ardevol, A. Enhancing PET Degrading Enzymes: A Combinatory Approach. *Chembiochem* **2024**, *25* (10), e202400084. DOI: 10.1002/cbic.202400084 From NLM Medline.

(66) Thapa, G.; Han, S. R.; Paudel, P.; Kim, M. S.; Hong, Y. S.; Oh, T. J. In Silico Analysis and Biochemical Characterization of Streptomyces PET Hydrolase with Bis(2-Hydroxyethyl) Terephthalate Biodegradation Activity. *J Microbiol Biotechnol* **2024**, *34* (9), 1836-1847. DOI: 10.4014/jmb.2404.04030 From NLM Medline.

(67) Zheng, Y.; Li, Q.; Liu, P.; Yuan, Y.; Dian, L.; Wang, Q.; Liang, Q.; Su, T.; Qi, Q. Dynamic docking-assisted engineering of hydrolases for efficient PET depolymerization. *ACS Catalysis* **2024**, *14* (5), 3627-3639. DOI: 10.1021/acscatal.4c00400.

(68) Zheng, Y.; Zhang, J.; You, S.; Lin, W.; Su, R.; Qi, W. Efficient thermophilic PET hydrolase enhanced by cross correlation-based accumulated mutagenesis strategy. *Bioresource Technology* **2024**, 130929. DOI: 10.1016/j.biortech.2024.130929.

(69) Avilan, L.; Lichtenstein, B. R.; König, G.; Zahn, M.; Allen, M. D.; Oliveira, L.; Clark, M.; Bemmer, V.; Graham, R.; Austin, H. P. Concentration-dependent inhibition of mesophilic PETases on poly (ethylene terephthalate) can be eliminated by enzyme engineering. *ChemSusChem* **2023**, *16* (8), e202202277. DOI: 10.1002/cssc.202202277.

(70) Gao, S.; Shi, L.; Wei, H.; Liu, P.; Zhao, W.; Gong, L.; Tan, Z.; Zhai, H.; Liu, W.; Liu, H. β-sheet Engineering of IsPETase for PET Depolymerization. *Engineering* **2024**. DOI: 10.1016/j.eng.2024.10.015.

(71) Jackering, A.; Gottsch, F.; Schaffler, M.; Doerr, M.; Bornscheuer, U. T.; Wei, R.; Strodel, B. From Bulk to Binding: Decoding the Entry of PET into Hydrolase Binding Pockets. *JACS Au* **2024**, *4* (10), 4000-4012. DOI: 10.1021/jacsau.4c00718 From NLM PubMed-not-MEDLINE.

(72) Mamtimin, T.; Ouyang, X.; Wu, W. M.; Zhou, T.; Hou, X.; Khan, A.; Liu, P.; Zhao, Y. L.; Tang, H.; Criddle, C. S.; et al. Novel Feruloyl Esterase for the Degradation of Polyethylene Terephthalate (PET) Screened from the Gut Microbiome of Plastic-Degrading Mealworms (Tenebrio Molitor Larvae). *Environ Sci Technol* **2024**, *58* (40), 17717-17731. DOI: 10.1021/acs.est.4c01495 From NLM Medline.

(73) Zhou, Y.; Zhang, J.; You, S.; Lin, W.; Zhang, B.; Wang, M.; Su, R.; Qi, W. High terephthalic acid purity: Effective polyethylene terephthalate degradation process based on pH regulation with dual-function hydrolase. *Bioresour Technol* **2024**, *413*, 131461. DOI: 10.1016/j.biortech.2024.131461 From NLM Medline.

(74) Schreiber, S.; Gercke, D.; Lenz, F.; Jose, J. Application of an alchemical free energy method for the prediction of thermostable DuraPETase variants. *Appl Microbiol Biotechnol* **2024**, *108* (1), 305. DOI: 10.1007/s00253-024-13144-z From NLM Medline.

(75) Qi, X.; Wu, Y.; Zhang, S.-T.; Yin, C.-F.; Ji, M.; Liu, Y.; Xu, Y.; Zhou, N.-Y. The unique salt bridge network in GlacPETase: a key to its stability. *Applied and Environmental Microbiology* **2024**, *90* (3), e02242-02223. DOI: 10.1128/aem.02242-23.

(76) Ogura, Y.; Hashino, Y.; Nakamura, A. Direct Screening of PET Hydrolase Activity in Culture Medium Based on Turbidity Reduction. *ACS Omega* **2024**, *9* (31), 34151-34160. DOI: 10.1021/acsomega.4c05488 From NLM PubMed-not-MEDLINE.

(77) Numoto, N.; Kondo, F.; Bekker, G.-J.; Liao, Z.; Yamashita, M.; Iida, A.; Ito, N.; Kamiya, N.; Oda, M. Structural dynamics of the Ca2+-regulated cutinase towards structure-based improvement of PET degradation activity. *International Journal of Biological Macromolecules* **2024**, *281*, 136597. DOI: 10.1016/j.ijbiomac.2024.136597.

(78) Miao, R.; Xu, G.; Ding, Y.; Ding, Z.; Woodard, J.; Tu, T.; Luo, H.; Wu, N.; Yao, B.; Guan, F.; Tian, J. Engineering dual-functional and thermophilic BMHETase for efficient degradation of polyethylene terephthalate. *Bioresour Technol* **2024**, *414*, 131556. DOI: 10.1016/j.biortech.2024.131556 From NLM Medline.

(79) Song, Y.; Li, A.; Cui, H.; Wu, L.; Zhou, B.; Li, X. Ancestral Sequence Reconstruction and Comprehensive Computational Simulations Unmask an Efficient PET Hydrolase with the Wobbled Catalytic Triad. *ChemSusChem* **2025**, *18* (10), e202402614. DOI: 10.1002/cssc.202402614 From NLM Medline.

(80) Wang, N.; Li, Y.; Zheng, M.; Dong, W.; Zhang, Q.; Wang, W. Unusual depolymerization mechanism of Poly(ethylene terephthalate) by hydrolase 202. *Chemosphere* **2025**, *372*, 144108. DOI: 10.1016/j.chemosphere.2025.144108 From NLM Medline.

(81) Wang, Z.; Zhang, J.; You, S.; Su, R.; Qi, W. Energy-guided accumulated mutation strategy achieves a highly efficient polyethylene terephthalate-degrading enzyme. *Biochemical Engineering Journal* **2025**, *219*, 109708. DOI: 10.1016/j.bej.2025.109708.

(82) Guo, X.; Xie, D.; Zhou, Y. QM/MM-MD Studies on the Degradation Mechanism and Size Effect of PET by PETase. *J Phys Chem B* **2025**, *129* (22), 5400-5410. DOI: 10.1021/acs.jpcb.5c00344 From NLM Medline.

(83) Lin, W.; Zheng, Y.; Zhang, J.; Zhou, Y.; Wang, M.; You, S.; Su, R.; Qi, W. Enhanced catalytic activity of polyethylene terephthalate hydrolase by structure-guided loop-focused iterative mutagenesis strategy. *J Hazard Mater* **2025**, *490*, 137837. DOI: 10.1016/j.jhazmat.2025.137837 From NLM Medline.

(84) Brown, D. K.; Penkler, D. L.; Sheik Amamuddy, O.; Ross, C.; Atilgan, A. R.; Atilgan, C.; Tastan Bishop, O. MD-TASK: a software suite for analyzing molecular dynamics trajectories. *Bioinformatics* **2017**, *33* (17), 2768-2771. DOI: 10.1093/bioinformatics/btx349 From NLM Medline.

(85) Qasim, L.; Alisaraie, L. ProS2Vi: a Python Tool for Visualizing Proteins Secondary Structure. *arXiv preprint arXiv:2408.03436* **2024**. DOI: 10.48550/arXiv.2408.03436.
